# A scalable double-barcode sequencing platform for characterization of dynamic protein-protein interactions

**DOI:** 10.1038/ncomms15586

**Published:** 2017-05-25

**Authors:** Ulrich Schlecht, Zhimin Liu, Jamie R. Blundell, Robert P. St.Onge, Sasha F. Levy

**Affiliations:** 1 Stanford Genome Technology Center, Stanford University, 3165 Porter Drive, Palo Alto, Calfornia 94304, USA; 2 Department of Biochemistry, Stanford University School of Medicine, Stanford, California 94305, USA; 3 Laufer Center for Physical and Quantitative Biology, Stony Brook University, Stony Brook, New York 11794-5252, USA; 4 Department of Biochemistry and Cellular Biology, Stony Brook University, Stony Brook, New York 11794-5215, USA; 5 Department of Applied Physics, Stanford University, Stanford, California 94305, USA

## Abstract

Several large-scale efforts have systematically catalogued protein-protein interactions (PPIs) of a cell in a single environment. However, little is known about how the protein interactome changes across environmental perturbations. Current technologies, which assay one PPI at a time, are too low throughput to make it practical to study protein interactome dynamics. Here, we develop a highly parallel protein-protein interaction sequencing (PPiSeq) platform that uses a novel double barcoding system in conjunction with the dihydrofolate reductase protein-fragment complementation assay in *Saccharomyces cerevisiae*. PPiSeq detects PPIs at a rate that is on par with current assays and, in contrast with current methods, quantitatively scores PPIs with enough accuracy and sensitivity to detect changes across environments. Both PPI scoring and the bulk of strain construction can be performed with cell pools, making the assay scalable and easily reproduced across environments. PPiSeq is therefore a powerful new tool for large-scale investigations of dynamic PPIs.

## Abstract (web summary)

Protein-protein interactions (PPIs) play a major role in defining biological functions. Here, the authors present PPiSeq, a method to quantitatively score dynamic PPIs in yeast combining fragment complementation assays, genomic double-barcoding, and an analytical framework to precisely call fitness from barcode lineage trajectories.

A genome-scale understanding of protein–protein interactions (PPIs) has been a long-standing goal of molecular and systems biology^1^. Several large-scale efforts have used the model yeast *Saccharomyces cerevisiae* to systematically catalogue all PPIs in a single environment using the yeast two-hybrid (Y2H) or the protein fragment complementation assay (PCA) systems^2^,^3^,^4^,^5^. These high-throughput screens take advantage of arrayed mating and differential colony growth on agar to identify PPIs. The resulting PPI networks have aided in defining the function of uncharacterized proteins, in characterizing protein complexes and modules that act in concert, and in identifying common functional network motifs^2^,^3^,^4^,^6^,^7^,^8^,^9^. Despite these advances, we are left with a static view of a cell. Unknown is how the protein interactome changes as cells differentiate or react to stress, even though these are each fundamental biological processes^10^. Also missing are PPIs that only occur in non-standard growth conditions.

Describing the global protein interactome dynamics, however, requires overcoming two main hurdles. First, higher throughput is required. *S. cerevisiae* has ∼6,000 genes, so measuring all pairwise PPIs in one environment requires ∼18 million strains to be assayed, each of which characterizes a single PPI. Observing how the protein interactome changes across perturbations would require re-assaying all ∼18 million strains for each perturbation studied. Even with recent advances such as high density yeast arrays and automated mating and scoring^11^, the time and reagent costs associated with performing screens across several environments make these studies impractical. Recently, a Y2H- and barcode-based pooled assay has been developed, which could potentially greatly increase the throughput of PPI detection^12^. However, because this method relies on plasmid-borne constructs under exogenous promoters, and because Y2H reporter activation requires nuclear localization, this platform is not ideal for characterizing how PPIs change under natural physiological conditions^13^.

Second, a graded PPI score is necessary to identify PPIs that change. In contrast with barcode-based Y2H, PCA uses genomically integrated fusion proteins under endogenous promoters with protein products interacting in their natural physiological setting, and therefore serves as a viable platform for detecting dynamic PPIs^14^,^15^. Many different protein fragment designs have been used with PCA, however the highest-throughput version uses a split murine dihydrofolate reductase (mDHFR) that, once reconstructed, allows colony growth on agar containing the *S. cerevisiae* DHFR inhibitor methotrexate (MTX). These assays have historically used colony size thresholds to call whether or not a PPI exists^4^. Graded scoring techniques that assign a fitness to each colony have been employed elsewhere, for example, in synthetic genetic interaction screens^16^. However, it is unclear if these techniques are applicable to PCA^17^. More recently, we and others have developed methods to detect dynamic PPIs via isolated growth of PCA strains in liquid multiwell plates followed by scoring of optical density growth curves^17^,^18^, a method that is capable of accurately detecting at least fourfold differences in the number of interacting protein molecules over a broad range of protein abundances^17^. However, the throughput of isolated liquid growth assays is inferior to colony-based assays, making their application to interactome-scale studies challenging.

A more scalable alternative is pooled growth of barcoded PCA strains followed by quantification to detect how changes in relative barcode abundance are altered across perturbations^18^. However, this technique is limited in two ways. First, a uniquely barcoded strain must be generated for each PPI, making interactome-scale barcoded libraries impractical to construct (for example, 18 million barcoded strains). Second, changes in relative barcode abundance are detected by comparing to a control condition, as is typical for barcode-based assays^12^,^19^,^20^,^21^. As we describe here, single time point relative barcode frequencies following growth are sensitive to the fitnesses of other cells in the pool, and therefore do not provide a robust score that can be compared across different pools or environments.

Here, we present a scalable and robust method to identify and quantitatively score dynamic PPIs that we call Protein–Protein interaction Sequencing (PPiSeq). The PPiSeq platform combines PCA, a new genomic double-barcoding technology, time-course barcode sequencing of competing cell pools, and an analytical framework to precisely call fitnesses from barcode lineage trajectories. We use these tools to examine the interactions between ∼100 protein pairs at high replication and across five environments. In a permissive environment, we find that the ability for PPiSeq to identify PPIs is on par with existing assays. In addition, PPiSeq finds that a large fraction of PPIs change across environments, many of which could be validated by other PPI assays. Finally, we show that bulk mating of barcoded strains can generate libraries exceeding 10^9^ double barcodes and therefore PPiSeq could potentially be used to simultaneously assay large networks containing thousands of PPIs.

## Results

### The PPiSeq platform

**Figure 1:**
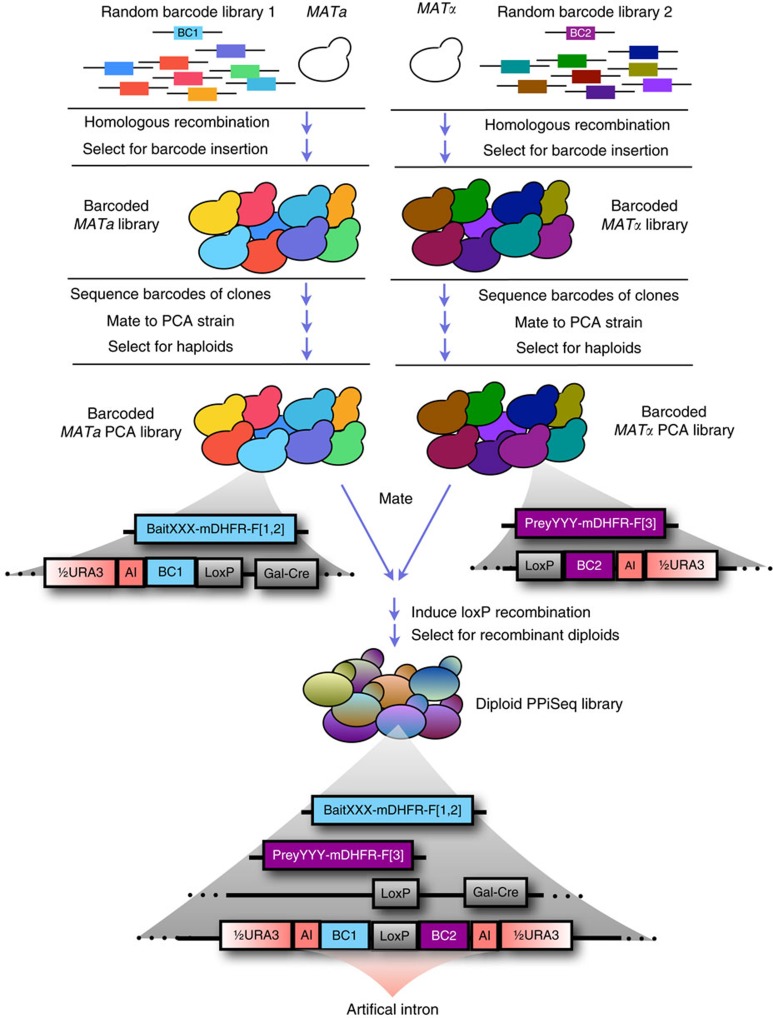
Construction of a PPiSeq library. Primers containing a random nucleotide barcode are inserted into a common genomic location of both MATα and MATa cells by homologous recombination, yielding large libraries of barcoded yeast cells. Clones from each library are picked at random and barcodes are identified by sequencing. Barcoded cells are mated to strains containing either a bait or prey protein fragment complementation construct^4^. Diploids are sporulated and haploids containing both a barcode and a PCA construct are selected. These haploids are mated to generate diploids that contain two barcodes and both bait and prey PCA constructs. Cre-induced loxP recombination brings the two barcodes to the same chromosome, and is selected for by reconstruction of a split *URA3* selectable marker^22^. The barcode-loxP-barcode sequence resides in an artificial intron and is not translated. Double barcodes mark which two PCA constructs are in each cell and are subsequently used as part of a sequencing-based pooled fitness assay to measure PPI scores.

We developed a general interaction Sequencing platform (iSeq). Barcodes that are adjacent to a loxP recombination site are introduced at a common chromosomal location in closely related *MATa* and *MATα* haploids. Barcodes are placed on opposite sides of the loxP site in each mating type such that mating and *Cre* induction causes recombination between homologous chromosomes, resulting in a *barcode-loxP-barcode* configuration on one chromosome (Fig. 1 and Supplementary Fig. 1). This event is selected for by loxP recombination-induced reassembly of a split *URA3* marker^22^. A double barcode unambiguously identifies both parents of a cross in highly complex cell pools, with each barcode half being in close enough proximity to allow the pair to be sequenced together by short-read sequencing. Next, double barcode strains are grown in pools, relative double barcode frequencies are assayed at several times, and their trajectories are used in combination with a global maximum likelihood method to estimate the relative fitness of each strain. Here, we use iSeq in combination with the DHFR PCA system to construct a PPiSeq platform (Fig. 1, Supplementary Fig. 1 and Supplementary Note 1)^4^.

### PPiSeq is accurate and highly reproducible

To test the reproducibility of PPiSeq and compare it to existing PPI assays, we selected nine bait (mDHFR-F[1,2]) and nine prey (mDHFR-F[3]) PCA strains and added five different barcodes to each. Bait (*IMD3, HOM3, SHR3, PRS3, DBP2, TPO1, DST1, FTR1* and *FMP45*) and prey (*IMD3, FPR1, SHR3, PRS3, DBP2, HXT1, PDR5, RPB9* and *SNQ2*) PCA constructs were chosen to encompass a number of previously discovered PPIs. We also added five different barcodes to two control strains that do not contain an mDHFR. Haploid barcoded PCA strains were next pairwise mated and pooled to generate a library of 2,500 double barcode (PPiSeq) strains, with each of the 100 genotypes being represented by 25 unique double barcodes.

**Figure 2:**
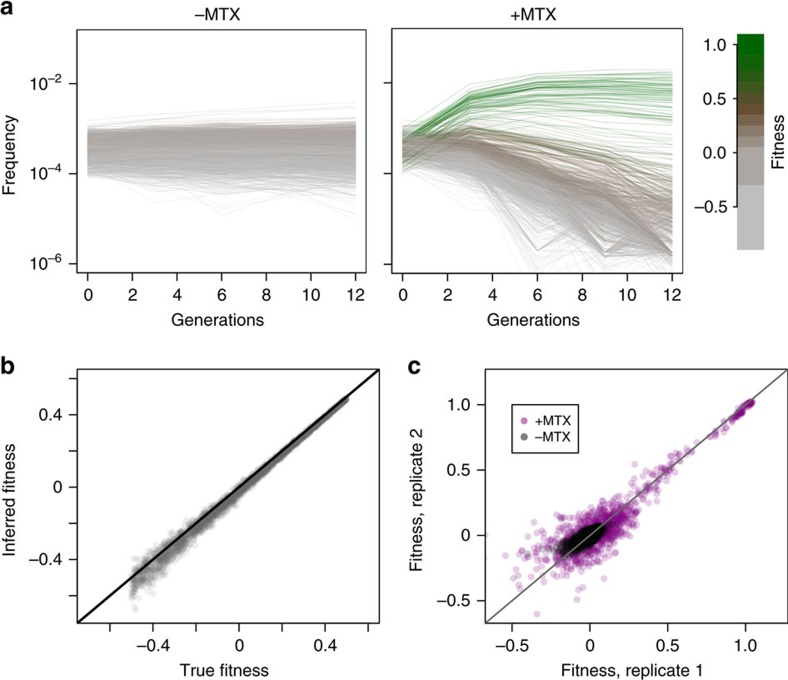
Lineage tracking and fitness estimation of double barcodes. (**a**) The frequency trajectories of 2,500 double barcoded PCA strains in the absence or presence of 0.5 μg ml^−1^ methotrexate (MTX). Frequencies are assayed every three generations during serial batch growth. Colour indicates the estimated fitness relative to strains in the same pool that lack mDHFR fragments. (**b**) Performance of fitness estimates on simulated data. Pearson's *r*=0.996. (**c**) Reproducibility of fitness estimates across growth replicates. Pearson's *r*>0.93 in MTX.

We next developed a pooled growth and bar-seq assay capable of robustly measuring the relative fitness of all strains in the pool. We expected that as low fitness PPiSeq strains drop out of the population, the frequency trajectories of a higher fitness strain will begin to ‘bend' as its competition gets tougher (green lines, Fig. 2a). The dynamics of this competition depends on the abundances and relative fitnesses of all strains in the pool^22^, and will therefore change if the composition of the pool changes. Because of this, barcode frequencies at a single time point do not provide a constant measure of fitness across conditions. We therefore monitored relative barcode frequencies over several early time points. We grew the PPiSeq pool in triplicate in standard yeast media and the presence or absence of a low concentration of MTX for ∼12 generations in serial batch culture, diluting 1:8 every 24 h (∼3 generations, Supplementary Fig. 2). To allow for fitness measurements of all strains, we chose a low concentration of MTX (0.5 μg ml^−1^, 400-fold lower concentration than traditional PCA^4^) where even strains lacking mDHFR will grow slowly. Double barcodes were sequenced at each dilution (every three generations). We found that reads representing putative PCR chimeras, double barcodes where each double barcode half stems from a different template, occurred at a low but predictable frequency (∼0.2%, Supplementary Note 2) and could confound our results. We therefore subtracted the expected number of PCR chimeras from each double barcode count and generated lineage trajectories with these corrected counts (Fig. 2a and Supplementary Data 1). In the absence of MTX, most PPiSeq strains do not change in frequency over time. However, in the presence of MTX, most strains are driven close to extinction by 12 generations, while others with higher fitness rise in frequency or have a slower decline. Higher fitness indicates that protein-mDHFR-fragment pairs within that strain interact to generate complete and functional mDHFR reporter proteins that, in turn, allow the strain to grow faster in the presence of low amounts of MTX.

To robustly calculate the fitness of each trajectory, we use a maximum likelihood strategy under a noise model that accounts for experimental errors (Supplementary Note 3)^22^. To make fitnesses comparable between replicates, or across different barcode pools or environments, we define a strain's fitness relative to the control strain that lacks any mDHFR fragments, whose fitness is set to zero. We find that this procedure performs extremely well on simulated data with parameters similar to our pooled growth experiments (Pearson's *r*=0.996, Fig. 2b and Supplementary Note 3 and 4), and across replicate growth experiments (Pearson's *r*>0.91 between all MTX(+) replicates, Fig. 2c). Fitness estimates are generally more accurate for higher fitness strains (those putatively identifying a PPI) because these trajectories are unlikely to fall to low frequencies where counting noise of sequencing reads will be high.

**Figure 3:**
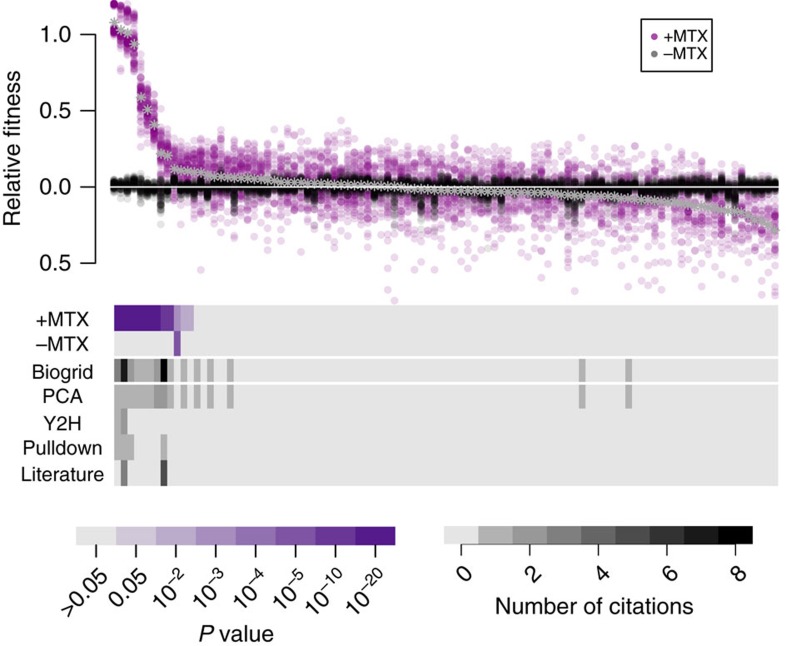
PPiSeq performance. Top: Relative fitnesses of each protein fragment pair grown in the absence (black) or presence (purple) of MTX. Each protein fragment pair is assayed with 25 unique double barcodes across 3 growth replicates for a total of ∼75 fitness estimates. Asterisks indicate the mean fitness of the protein fragment pair in MTX across all measurements and PPIs are ranked according to this fitness. Bottom purple: Heat map of the significance of the fitness difference between each protein fragment pair and control strains in the same pool that lack mDHFR fragments. *P* values are calculated using a Bonferroni-corrected Student's *t*-test. Bottom grey: the number of times each protein-protein interaction has previously been cited. Biogrid is the sum of all forms of evidence: protein fragment complementation (PCA), yeast two-hybrid (YTH), pull down/mass spectroscopy (Pulldown), and low-throughput studies (Literature).

We compared the fitness for each PPI across all ∼75 replicate estimates (∼25 double barcodes per PPI, 3 replicate growth experiments) in the presence or absence of MTX (Fig. 3 and Methods). Standard errors on fitness are low (typically, s.e.m.<0.05 in MTX(+), Supplementary Fig. 3), with higher fitness PPIs having the lowest errors (s.e.m.<0.02 in MTX(+) for PPIs with fitness >0.07). We next compared the fitness values of each PPI against the fitness values of the control strains lacking mDHFR in both MTX(+) and MTX(−) conditions (Fig. 3 and Supplementary Data 2). As expected in MTX(−), almost none of the strains differ significantly in fitness from the control. The single exception is Prs3-F[1,2]:Fpr1-F[3], which displayed a small but highly significant fitness advantage (fitness=0.04, *P* value <2 × 10^−6^, Bonferroni corrected one-sided Student's *t*-test) that is perhaps due to an adaptive mutation that occurred in the parental PCA strain before barcoding. After removing the Prs3:Fpr1 strain from consideration, we find 11 significant PPIs in MTX(+), 10 that have been previously identified^4^,^23^, and one that is new, Ftr1:Pdr5 (fitness=0.10, *P* value <0.002, Supplementary Data 2). We validated Ftr1:Pdr5 by two additional assays. First, we tracked the optical density (OD600) of Ftr1:Pdr5 PPiSeq strains and the mDHFR(−) control strains grown in isolation in MTX(+) media^17^,^18^ and found that Ftr1:Pdr5 strains rise in optical density faster (*P* value <2 × 10^−11^, Student's *t*-test, Supplementary Fig. 4)^18^. Second, we performed a less sensitive Renilla luciferase (Rluc) PCA^24^ and found that Ftr1:Pdr5 has a consistently higher (but not significant) luminescence when compared to control cells (*P* value=0.24, Student's *t*-test). As discussed below, the Rluc PCA finds a significant Ftr1:Pdr5 interaction in an alternative environment (*P* value <0.02 in 200 μM copper sulfate, Student's *t*-test), strongly suggesting that our finding in this permissive environment is not a false positive.

Our PPiSeq assay missed five putative PPIs that had been discovered by traditional PCA^4^. Three (Shr3:Hxt1, Tpo1:Snq2, and Fmp45:Pdr5) showed elevated but not significant fitness increases in MTX(+) (0.10, 0.08, and 0.06, respectively). As discussed below, PPiSeq does find all of these interactions to be significant in at least one perturbation environment, suggesting that these PPIs are sensitive to the environment and that environmental differences between PPiSeq and traditional PCA may impact their detection. The remaining two PPIs (Fmp45:Snq2 and Tpo1:Shr3) could not be detected by PPiSeq in any environment, but could be validated as being PPIs using isolated growth and optical density tracking over 32 h of growth (Supplementary Fig. 5). Notably, differences in optical density between Tpo1:Shr3 and control strains only began to appear around 25 h of growth, likely caused by a change in Tpo1 localization following the diauxic shift^25^,^26^, suggesting that our current 24 h growth-bottleneck regime is not sensitive to PPIs that are specific to this later growth phase and that longer growth-bottleneck cycles may capture additional PPIs.

Overall, the ability of PPiSeq to detect PPIs appears to be on par with existing PPI assays; in this test set, PPiSeq discovered 10 of 15 PPIs that have been described by other assays, 1 new PPI validated here, and no additional spurious PPIs. When considering other environments, PPiSeq discovered 14 of 16 PPIs that have been discovered here or elsewhere. However, in contrast with previous high-throughput assays, detected PPIs span a reproducible range of positive fitnesses. Growth rate of PCA strains in MTX has previously been found to correlate with the number of functional mDHFR molecules per cell^17^,^18^, suggesting that fitness differences in our assay are founded in differences in the abundance, localization, or binding of the interacting proteins.

### PPiSeq detects dynamic PPIs

**Figure 4:**
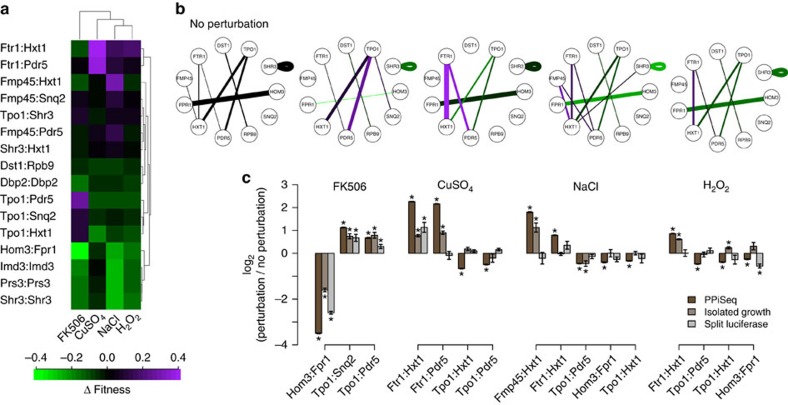
Dynamic PPIs. (**a**) Heatmap of PPIs across environments. All PPIs discovered here or elsewhere in standard conditions are shown. Colors are the fitness in each condition minus the fitness in the permissive condition. Cells are arranged by unsupervised hierarchical clustering. (**b**) PPI network plots of PPIs across five environments. Proteins that only interact with self are omitted. Colours are as in **a**. Edge width is proportional to the fitness and only significant edges are shown. PPIs expected under the standard condition are listed in **a**. (**c**) Barplots of the log ratio of the interaction score of a perturbation over the interaction score in the permissive environment as detected by three assays: PPiSeq (dark brown), split mDHFR clonal growth dynamics (light brown), and split Renilla luciferase luminescence (grey). Error bars are s.e.m. *, p<0.05, Student's *t*-test against the permissive environment.

One advantage of using a pooled growth and bar-seq approach for detecting PPIs is that, once a barcoded PCA pool is constructed, it is trivial to re-test the entire interaction space across perturbations in order to detect PPIs that are dynamic. Here, we grew the pool of 2,500 PPiSeq strains in triplicate in MTX(−) and MTX(+) media supplemented with one of four additional perturbagens: 0.001% hydrogen peroxide (oxidative stress), 175 mM sodium chloride (high salt), 200 μM copper sulfate (high copper), and 50 μM of FK506, an inhibitor of calcineurin function in yeast. We calculated the fitness of each strain in each environment relative to the mDHFR(−) control strain using the maximum likelihood strategy described above. As expected, we found major fitness differences between strains within each MTX(+) environment, but not within the MTX(−) environments (Fig. 4, Supplementary Fig. 6, and Supplementary Data 4). Surprisingly, 86% of detected PPIs significantly changed in fitness in at least one perturbation relative to the permissive environment (12 of 14, *P*<0.05, Bonferroni corrected Student's *t*-test, Supplementary Data 4) and 50% were undetectable by our assay in at least one environment (7 of 14, *P*>0.05, Bonferroni corrected one-sided Student's *t*-test, Supplementary Data 5). To validate these changes, we next selected 16 PPI-environment combinations where fitness was significantly different from the permissive environment, and assayed each by both optical density tracking and Rluc PCA (Fig. 4c). We found that 9 of 16 dynamic PPIs could be validated by at least one method.

A number of factors appear to underlie PPI changes across environments. One expected change is the interaction between the aspartate kinase Hom3 and the peptidyl-prolyl cis-trans isomerase Fpr1 in FK506, which has been previously found to physically disrupt this interaction^27^. Consistent with our previous work^18^, we find that the fitness of the Hom3:Fpr1 PPiSeq strain is diminished ∼10-fold in FK506 (*P*<10^−59^, Supplementary Data 3 and 4). Other dynamic PPIs appear to be due, at least in part, to changes in protein expression. For example, many of the changes that we detect—increased fitness of Tpo1:Pdr5 and Tpo1:Snq2 in FK506, Ftr1:Hxt1 and Ftr1:Pdr5 in copper, and Hxt1:Fmp45 in high salt—co-vary with changes in mRNA expression of one or both interacting partners that are reported in the literature^18^,^28^,^29^,^30^,^31^. Still other dynamic PPIs may be due to changes in protein localization. For example, fitness of the Tpo1:Pdr5 PPiSeq strain decreases in high salt (*P*<10^−11^), even though both *TPO1* and *PDR5* have been shown to increase in mRNA expression (4.7- and 2.7-fold, respectively^32^). This contradiction appears to be resolved by the finding that Pdr5, but not Tpo1, becomes depleted from the plasma membrane in high salt (Supplementary Fig. 7, Supplementary Note 5)^33^.

### PPiSeq is scalable

We have previously shown that at least 500,000 uniquely barcoded strains can be tracked in parallel in a single cell pool^22^. Furthermore, we found that for the majority of barcodes, errors in frequencies are consistent with counting noise stemming from finite read depths, rather than some other factor in the experimental protocol (Supplementary Note 4). Given exponentially declining sequencing costs^34^, it is therefore possible that several million double barcodes could be assayed in parallel. We reasoned that in order for our PPiSeq platform to reach these scales, two criteria must be met. First, PPiSeq must be capable of generating a large number of double barcode strains by pooled mating. Although it is technically possible to probe extremely large interaction spaces by pairwise mating in ordered arrays (for example, ref. 4), the cost and time required to do so is high, and this requirement would greatly reduce the flexibility and scalability of the platform. Second, the distribution of initial double barcode frequencies must be of a form that allows the fitness of most strains in the pool to be measured at reasonable sequencing depths. A distribution where many double barcodes are missing or are present at low frequencies would result in a large fraction of uncharacterized interactions.

**Figure 5:**
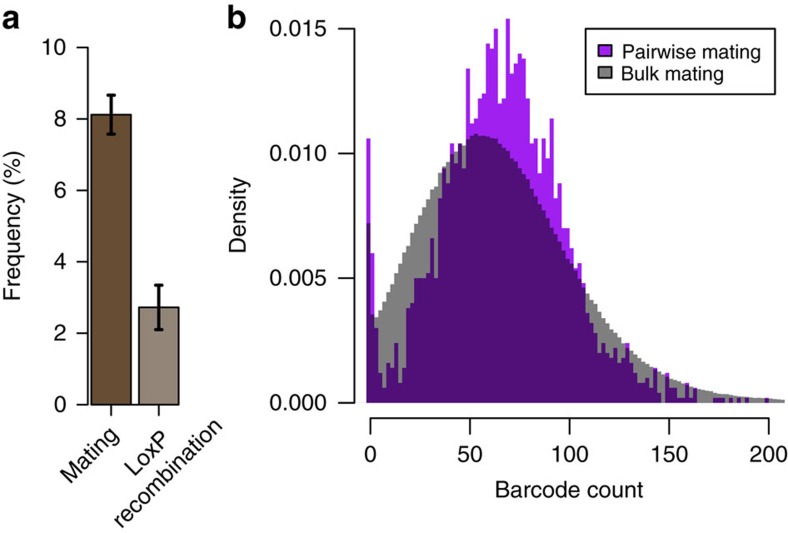
PPiSeq is scalable. (**a**) Lower bounds of the mating and loxP recombination efficiencies of a pooled mating and recombination protocol that uses ∼10^10^ cells per standard plate. Error bars are s.e.m. Each plate has the potential to generate >2 × 10^7^ double barcodes. (**b**) Density plot of the frequencies of ∼10^6^ double barcodes that were generated by bulk mating (grey) and 2,500 double barcodes that were generated by pairwise mating (purple). In both cases, the average number of reads per barcode is 67.

To test how many unique double barcodes could be realistically generated by pooled mating, we developed a protocol that mates ∼10^10^ haploids on a standard agar plate, and then selects for diploid double barcode recombinants (Supplementary Methods). On the basis of experimental tests, we estimated the lower bounds of the frequency of mating (8%) and loxP recombination (2%) of this protocol, and predicted that at least 2 × 10^7^ (i.e. 10^10^ × 8.1% × 2.7%) unique double barcoded diploids are generated per plate (Fig. 5a and Supplementary Note 6). Based on this performance, we estimate that double barcode library sizes exceeding 10^9^ could be achieved by a single investigator (∼50 mating plates).

We next compared the initial double barcode frequency distribution of a large bulk mating (∼1 million double barcodes possible across 5 mating plates) to the smaller pairwise mating we used to generate the PPiSeq strains above (2,500 double barcodes possible), and found that the two protocols resulted in similar barcode frequency distributions (Fig. 5b). At an average sequencing depth of ∼67 reads/barcode (Supplementary Note 7), bulk and pairwise mating protocols detect a similar number of double barcodes at low and moderate frequencies (>98% at >1 read, >95% at >10 reads), suggesting that even moderate read depths will be sufficient to characterize most double barcodes in the pool.

## Discussion

We describe a highly parallel PPiSeq assay that is sensitive, accurate, and graded. Importantly, PPiSeq provides a quantitative score (fitness) for each PPI that is robust to changes in the environment or pool constituents. To probe larger interaction sets with PPiSeq, large barcode libraries must first be generated and mated to individual PCA strains. However, we have recently shown that libraries of thousands of barcoded yeast can be generated in parallel using our iSeq platform and Illumina sequencing^35^, and these barcodes can be easily added to PCA strains using the synthetic genetic array technology^11^,^36^. Furthermore, once barcoded PCA libraries have been generated, both double barcode library construction and fitness assays can be performed in large cell pools. PPiSeq is therefore a powerful new platform for large-scale investigations of dynamic PPIs.

The growth of each PCA strain is known to correlate with the number of reconstituted mDHFR reporter proteins per cell^15^,^17^,^37^, which, in turn could be influenced by several factors including the abundance of each interacting protein, the binding affinity, and the extent of co-localization of each binding pair. Protein abundances appear to have a large influence on fitness. For the 16 PPIs in our test set, fitness correlates reasonably well with the abundance of the least abundant interaction partner (Spearman's rho=0.68, Supplementary Fig. 8)^38^. Additionally, many of the changes in fitness across environments that we detect co-vary with changes in mRNA expression of one or both interacting partners that are reported in the literature. However, other factors are likely to be important as well. For example, a recent proteome-wide screen found that nearly as many proteins change in localization as change in abundance when cells are exposed to hydroxyurea (28 and 40, respectively)^39^. In our test set, we find one example where a change in localization appears to be driving a PPI change (Tpo1:Pdr5). However, we do caution that an unbiased and systematic characterization of the factors underlying dynamic protein interaction will require combining PPiSeq with genome-scale mRNA abundance, protein abundance, and protein localization studies under the same conditions.

For cells treated with FK506, PPiSeq not only detects a change in the PPI target of the drug, Hom3:Fpr1, but also changes in other PPIs such as Tpo1:Snq2 and Tpo1:Pdr5. In this case, additional changes appear to be caused by a specific cellular response to the drug, as each of these proteins are efflux transporters^18^. However, dynamic PPIs that are a response to global changes in the cell physiology or that are due to off-target binding of a drug may also be likely. Avoiding off-target effects, as well as a systems level understanding of a drug's effect on the cell, are often the primary concerns of drug development^40^,^41^,^42^. Because of the ease by which large numbers of PPIs can be quantitatively screened across many perturbations in relatively small volumes of media, PPiSeq therefore provides a powerful new tool for high-throughput drug screening.

More generally, iSeq provides a new framework for performing large-scale dynamic interaction screens. Because PPI scoring and the bulk of strain construction can be performed in cell pools, instead of one-by-one, a major throughput limitation to interaction screens has been removed. Furthermore, iSeq can in theory be used to investigate combinations of any two genetic elements in yeast, such a gene knockouts or engineered constructs, and is therefore likely to have broad utility beyond PPI screens.

## Methods

### Pairwise diploid PPiSeq library construction

PPiSeq haploids (Supplementary Note 1) were systematically mated to create 50 × 50=2,500 diploid strains using standard protocols on a Singer ROTOR HDA robot. Diploid strains were selected on YPD+nourseothricin+hygromycin B. Expression of the Cre-recombinase and strains that successfully recombined their loxP sites were then selected on CSM-uracil+galactose media. A frozen stock of the pool was created by washing the 2,500 strains off the agar plates using YPD+15% glycerol and storing aliquots at −80 °C.

### Pooled growth assays

An aliquot of the frozen pairwise-mated double barcoded PCA pool was thawed and grown overnight by inoculating 200 μl into 20 ml of YNB+ammonium sulfate+dextrose+histidine+leucine. At late log phase (OD600=1.89), four aliquots of 1 ml each were collected, pelleted by centrifugation, and stored as time-0 samples at −80 °C. A 48-well plate was then inoculated with YNB+ammonium sulfate+dextrose+histidine+leucine media (700 μl) with or without 0.5 μg ml^−1^ methotrexate and the pool at a starting OD600=0.0525. The media was supplemented with one of the following components: DMSO (final at 0.5%), FK506 (final at 50 μM), hydrogen peroxide (final at 0.001%), sodium chloride (final at 175 mM), or copper sulfate (final at 200 μM). Every condition was assayed in triplicate. Every 3 generations (that is, at 3, 6, 9 and 12 pool generations), 600 μl were collected, pelleted by centrifugation and then stored at −80 °C. 70 μl were inoculated into fresh media of the same type (that is, with or without methotrexate and containing the same component). Genomic DNA was then extracted from all 124 samples using the YeaStar Genomic DNA Kit (Zymo Research), and double barcodes were PCR-amplified using the Q5 High-Fidelity 2X Master Mix (NEB) according to manufacturer instructions. PCR was performed with barcoded up and down sequencing primers (multiplexing tags, see Data File 5) that produce a double index to uniquely identify each sample. PCR products were confirmed by agarose gel electrophoresis. After PCR, samples were combined and bead cleaned with Thermo Scientific Sera-Mag Speed Beads Carboxylate-Modified particles. Sequencing was performed on an Illumina HiSeq 2500 with 25% PhiX DNA. The PhiX DNA was necessary to increase the read complexity for proper calibration of the instrument (see ref. 22).

### Double barcode sequence analysis

Barcode reads were processed with custom written software in Python and R as described^22^, with modifications. Briefly, sequences were parsed to isolate the two barcode regions (38 base pairs each), sorted by their multiplexing tags (see above), and removed if they failed to pass any of three quality filters: (1) The average Illumina quality score for both barcode regions must be greater than 30, (2) the first barcode must match the regular expression ′\D*?(.ACC|T.CC|TA.C|TAC.)\D{4,7}?AA\D{4,7}?AA\D{4,7}?TT\D{4,7}?(.TAA|A.AA|AT.A|ATA.)\D*|\D*?5′-GTACTAACGGCTAATTTGGTGCC;CA-3′\D*′, and 3) the second barcode must match the regular expression ′\D*?(.TAT|T.AT|TT.T|TTA.)\D{4,7}?AA\D{4,7}?AA\D{4,7}?TT\D{4,7}?(.GTA|G.TA|GG.A|GGT.)\D*′. A BLAST database containing all expected double barcodes (76 bases each) was constructed and each read was blasted (word size=11, reward=1, penalty=−2) against this database. Double barcode reads that blasted at an e<10−28 (∼2 mismatches) to an expected double barcode were summed to calculate as an initial estimate of the read number of each double barcode in each condition.

### Comparisons to existing PPI studies

Interaction data was downloaded from the Biogrid on 1 December 2015 (*S. cerevisiae* version 3.4.131)^23^,^43^. PPIs we sorted based on the form of evidence: Protein Fragment Complementation (PCA)^4^,^18^,^44^, Yeast Two Hybrid (YTH)^3^,^5^, Affinity Pull-Down Assays (Pulldown)^45^,^46^,^47^,^48^, and other lower-throughput methods in the literature (Literature).

### Significance test for dynamic PPIs

The fitness of each double barcode strain in each environment was determined as described in Supplementary Note 3. Fitnesses for a given PPI were compared across environments using a two-sided Student's *t*-test Bonferroni corrected for 400 tests.

### PPI scoring by isolated growth optical density dynamics

Haploid PCA strains^4^ were streaked from frozen stocks onto YPD to recover isolated colonies. *MATa* PCA strains harbouring BAIT-DHFR-F[1,2]-NatMX were mated one-by-one to *MATα* PREY-DHFR-F[3]-HphMX PCA strains in YPD liquid media. A control diploid strain that lacks DHFR was generated by mating a barcoded *MATa* ho::NatMX strain with a barcoded *MATα* ho::HphMx strain. Following 12 h of mating, cells were plated onto YPD+nourseothricin+hygromycin B agar and grown for 48 h at 30 °C to select for diploids. One colony of each diploid was inoculated into YPD+nourseothricin+hygromycin B liquid media, grown for 12 h at 30 °C, and then stored in 15% glycerol at −80 °C. Cells were streaked from frozen stocks onto YPD and grown for 48 h at 30 °C. Three isolated colonies of each strain were suspended in sterilized water and counted. For each replicate, 6.4 × 10^4^ cells were inoculated into 150 μl of media in black-walled, clear-bottom 96-well plates (Nunc #265301). Media was synthetic dextrose supplemented with standard concentrations of the amino acids histidine, leucine, and uracil, plus methotrexate (0.5 μg ml^−1^) and one of the following perturbagens: DMSO (final at 0.5%), FK506 (final at 50 μM), hydrogen peroxide (final at 0.001%), sodium chloride (final at 175 mM), or copper sulfate (final at 200 μM). Plates were sealed with foil (Costar # 6570) and shaken at 1,300 r.p.m. (DTS4, Elmi) at 30 °C. The optical density (OD units at 600 nm) of each microwell culture was recorded (F500, Tecan) at 0, 8, 10, 12, 14, 16, 18, 20, 22, 24, and 32 h. The area under the curve (AUC) was calculated as the sum of all OD readings before saturation (32 h) for each strain in each environment. The relative fitness for a strain in a specific condition was quantified with following equation: (AUC_target strain_−AUC_control strain_)_condition_/(AUC_target strain_−AUC_control strain_)_DMSO_.

### Luciferase protein fragment complementation assay

To construct Renilla luciferase (Rluc) PCA strains, we replaced the DHFR fragments with Rluc PCA fragments in haploid DHFR PCA strains^4^ via homologous recombination. The Rluc-F[1]-NatMX homologous recombination cassette was PCR amplified from the pAG25-linker-Rluc-F[1]-NatMX plasmid^24^, and the Rluc-F[2]-HphMX cassette was PCR amplified from the pAG32-linker-Rluc-F[2]-HphMX plasmid^24^. We used the same pair of primers for the amplification of both homologous recombination cassettes. The forward primer (5′-GGCGGTGGCGGATCAGGAGGC-3′) anneals to the linker sequence in pAG25-linker-Rluc-F[1]-NatMX or PAG32-linker-Rluc-F[2]-HphMX. The reverse primer (5′-TTCGACACTGGATGGCGGCGTTAG-3′) anneals to the 3′-end of the TEF terminator region of NatMX or HphMX. To increase the recombination efficiency for some genes, it was necessary to add an additional 40 bp to the forward primer that matches gene-specific sequence upstream of the stop codon. In all cases, *MATa* PCA (DHFR-F[1,2]-NatMX) strains were transformed with the Rluc-F[2]-HphMX cassettes and *MATα* PCA (DHFR-F[3]-HphMX) strains were transformed with the Rluc-F[1]-NatMX cassettes. Transformants were selected by plating on YPD plus the appropriate antibiotic, and proper incorporation of the Rluc PCA cassette was validated by PCR. Next, *MATa* PCA strains harbouring BAIT-Rluc-F[1]-NatMX were mated one-by-one to *MATα* PREY-Rluc-F[2]-HphMX strains in YPD liquid media. Following 12 h of mating, cells were plated onto YPD+nourseothricin+hygromycin B agar and grown for 48 h at 30 °C to select for diploids. One colony of each diploid was inoculated into YPD+nourseothricin+hygromycin B liquid media, grown for 12 h at 30 °C, and then stored in 15% glycerol at −80 °C.

Triplicate fresh colonies of each diploid Rluc PCA strain were grown in 5 ml synthetic dextrose media supplemented with standard concentrations of histidine, leucine, and uracil at 30 °C for 24 h, then diluted 1:32 into 5 ml of the same media supplemented DMSO (0.5%), FK506 (50 μM), hydrogen peroxide (0.001%), sodium chloride (175 mM), or copper sulfate (200 μM). Cells were grown for 24 h at 30 °C, diluted 1:32 again into fresh media containing the same supplement, and grown for another 6 h. Cells were counted, and 1–2 × 10^7^ cells were pelleted, and resuspended in 180 μl phosphate-buffered saline (PBS), pH 7.2 containing 1 mM EDTA. Cells were transferred to white 96-well flat bottom plates (Greiner bio-one # 655075). The luciferase substrate, benzyl coelenterazine (Nanolight #301), was diluted 1:10 from the stock (2 mM in absolute ethanol) using 1 × PBS, and 20 μl of diluted substrate was added to each sample (to a final concentration of 20 μM). A Centro LB 960 microplate luminometer (Berthold Technologies) was used to measure the Rluc PCA signal, which was integrated for 10 s. Changes in luminescence in response to a specific condition were calculated by the following equation: luminescence_condtion_/luminescence_DMSO_.

### Pooled construction of a large double barcode library

iSeq-barcoded haploid *MATa* (1137 SHA345+BC strains) and *MATα* (844 SHA349+BCs strains) strains were grown to saturation (48 h at 30 °C) in 100 μl YPD+G418 in 96-well plates. Clones of the same mating type were pooled to generate the *MATα* and *MATa* barcode pools, and stored in 15% glycerol aliquots at −80 °C. The frozen barcode pools were thawed completely at room temperature, and 1.35 × 10^9^ cells of the *MATα* pool and 2.9 × 10^9^ cells of *MATa* pool were each inoculated into 200 ml YPD+G418 and grown for 20 h at 30 °C. A cell count of each pool was taken, the two pools were combined at equal cell densities, and this mixed pool was streaked onto six YPD plates at a density of 10^10^ cells per plate to mate. Cells were grown on YPD for 24 h at 30 °C, and then all plates were scraped and pooled in water. The number of cells in this pool was counted and ∼3.3 × 10^10^ cells (⅓ of all the cells) were plated onto 30 SC-Met-Lys plates at equal cell densities. Cells were incubated for 48 h at 30 °C and then replicated onto another 30 SC-Met-Lys plates. After another 48 h incubation at 30 °C, cells were scraped from the 30 SC-Met-Lys plates and pooled in water. All the cells (4.2 × 10^10^) were spun down, resuspended with 1 L SC+Gal - Ura, and grown for 48 h at 30 °C. Then cells were counted and 100 ml (∼8.2 × 10^9^ cells) was inoculated into 1 L SC-Ura media and grown for 48 h at 30 °C to further enrich for loxP recombinants. Finally, all the cells were collected to form the pooled diploid barcode library.

### Sequencing of bulk mated double barcode pools

Genomic DNA of the pooled diploid barcode library was extracted using the MasterPure Yeast DNA Purification Kit (epicentre #MPY80200). To completely remove RNA, an extra RNase treatment, DNA precipitation with isopropanol, and washing with 70% ethanol were added after the recommended protocol from the manufacturer. Double barcode amplicons were generated using a two-step PCR protocol^22^. Briefly, a 5-cycle PCR with OneTaq polymerase (New England Biolabs) was performed in 60 reactions for the large double barcode library, amplifying ∼1,000 copies per unique lineage tag. The PCR products were then pooled and purified with PCR Cleanup columns (Qiagen) at six PCR reactions per column. A second 23-cycle PCR was performed with high-fidelity PrimerSTAR Max plolymerase (Takara) in 30 reactions, with 15 μl of cleaned product from the first PCR as template and 50 μl total volume per tube. PCR products from all reaction tubes were pooled and purified using a PCR Cleanup column (Qiagen) and eluted into 50 μl of water. The appropriate PCR band was isolated by E-Gel agarose gel electrophoresis (Life Technologies) and quantitated by Qubit fluorometry (Life Technologies). Sequencing was performed on an Illumina HiSeq 2500 with 25% PhiX DNA spike-in. The PhiX DNA was necessary to increase the read complexity for proper calibration of the instrument (see ref. 22).

### Code availability

All python scripts used to estimate lineage fitness are available from the corresponding author on reasonable request.

### Data availability

All barcode sequences and counts for the PPiSeq assays are available in Supplementary Data 1. Additional data sets generated and/or analysed during the current study are available from the corresponding author on reasonable request.

## Additional information

**How to cite this article:** Schlecht, U. *et al*. A scalable double-barcode sequencing platform for characterization of dynamic protein-protein interactions. *Nat. Commun.* **8**, 15586 doi: 10.1038/ncomms15586 (2017).

Publisher's note: Springer Nature remains neutral with regard to jurisdictional claims in published maps and institutional affiliations.

## Supplementary Material

Supplementary Information Supplementary Figures, Supplementary notes, and Supplementary References

Supplementary Data 1 Count trajectories of each strain in each growth condition. Column headers: "PPI_pair" is a unique identifier for each PPI. "barcode_pair" is a unique identifier for each barcode pair. "Bait" is the bait PCA construct. "Prey" is the prey PCA construct. "Bait_barcode" is the barcode that marks the bait (5 for each bait PCA construct). "Prey_barcode" is the barcode that marks the prey (5 for each prey PCA construct). "Condition" is the growth condition (e.g. DMSO or FK506). "Methotrexate_selection" indicates if methotrexate was included in the growth media. "Replicate" indicates the growth replicate (1,2,3). "Reads_XX_generations" is the count of the number of sequencing reads of the double barcode pair at a given time in the pooled growth experiment. "Reads_XX_generations_corrected" is the count of the number of sequencing reads of the double barcode pair after removing the the number of reads that would be expected by putative PCR chimeras. "Fitness" is the fitness determined by Poisson likelihood maximization strategy. "Likelihood" is a measure of how well the observed trajectory fits the projected trajectory from the estimated fitness (higher is a better fit). "Trajectory_used" is a logical value on whether the fitness was used for downstream comparisons. Those with low likelihoods are not used.

Supplementary Data 2 PPIs in the benign DMSO condition. Column headers: "bait" is the bait PCA construct. "prey" is the prey PCA construct. "fitness +MTX" is the fitness in media containing methotrexate relative to the ho::NatMX/ho::HphMX strains. "fitness -MTX" is the fitness in media lacking methotrexate relative to the ho::NatMX/ho::HphMX strains. "Bonferroni corrected p-value +MTX" is the a one sided t-test of fitness values in media containing methotrexate versus the ho::NatMX/ho::HphMX control strain corrected for 500 tests. "Bonferroni corrected p-value -MTX" is the a one sided t-test of fitness values in media lacking methotrexate versus the ho::NatMX/ho::HphMX control strain corrected for 500 tests. "Biogrid" is the number of times a PPI has been previously discovered across all data sources. "PCA" is the number of times a PPI has been previously discovered by protein fragment complementation. "Y2H" is the number of times a PPI has been previously discovered by yeast two-hybrid. "Pulldown" is the number of times a PPI has been previously discovered by pull-down mass spectrometry. "Literature" is the number of times a PPI has been previously discovered by additional low-throughput methods.

Supplementary Data 3 The mean fitness of each PPI in each environment in media containing methotrexate relative to the ho::NatMX/ho::HphMX strains.

Supplementary Data 4 P-values of two sided t-tests of fitness values in each perturbation versus the same PPI in the benign DMSO environment, Bonferroni corrected for 400 tests.

Supplementary Data 5 P-values of one sided t-tests of fitness values in each environment in media containing methotrexate versus the ho::NatMX/ho::HphMX control strain, Bonferroni corrected for 500 tests.
