# Supplementary material for: A scalable double-barcode sequencing platform for characterization of dynamic protein-protein interactions
Source: Nat Commun. 2017 May 25;8:15586. doi: 10.1038/ncomms15586 (PMC5458509; doi:10.1038/ncomms15586)
Supplement: Supplementary Information — Supplementary Figures, Supplementary notes, and Supplementary References [file ncomms15586-s1.pdf]

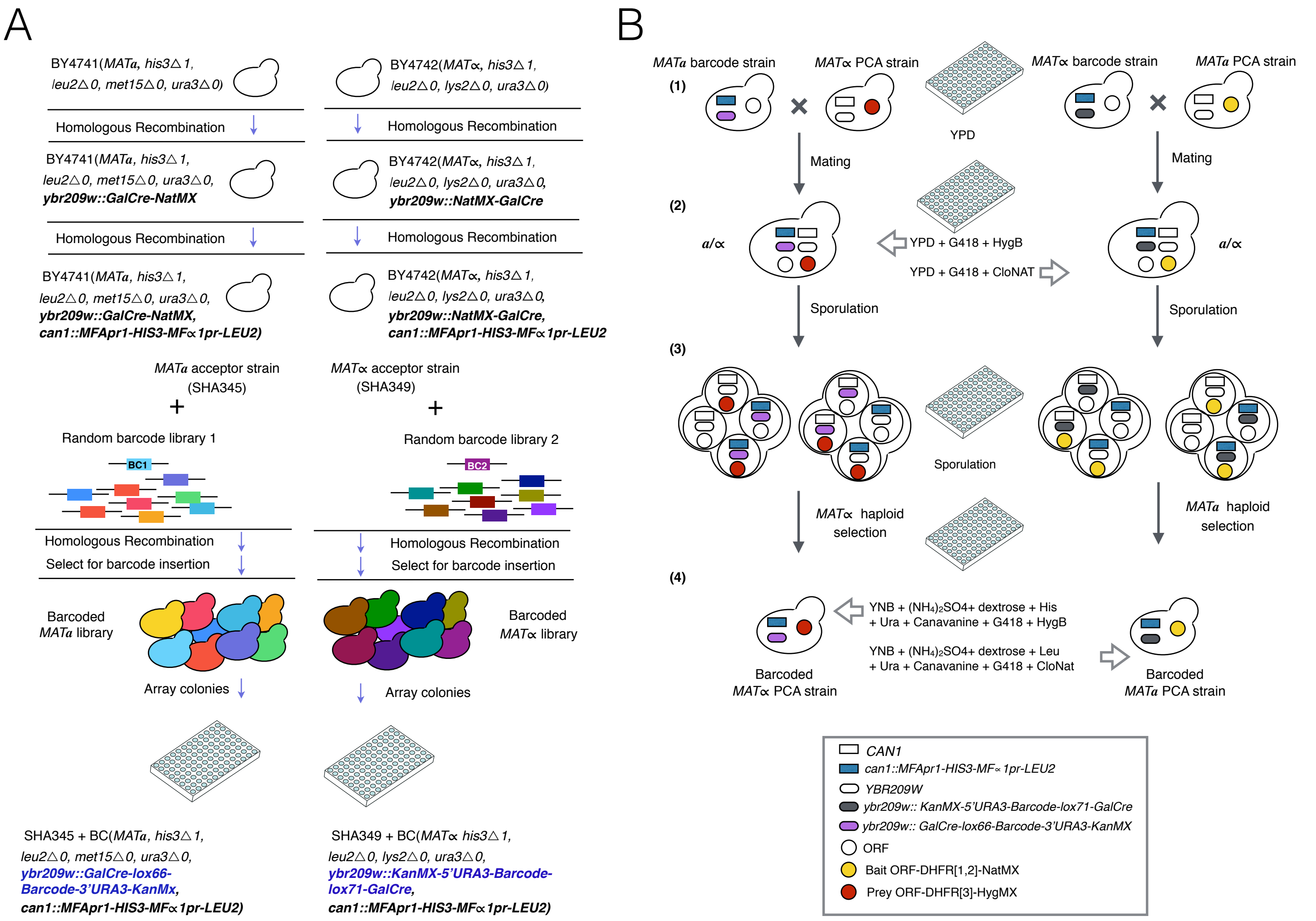

Supplementary Figure 1. Strain construction for Protein-Protein interaction Sequencing (PPiSeq). (A) Construction of barcoded yeast libraries. Gal-Cre-NatMX was inserted into the YBR209W locus of BY4741 and BY4742 strains in opposite orientations via homologous recombination. Then, the CAN1 locus in two strains was replaced with the dual magic marker (MFApr1-HIS3-MF∝1pr-LEU2) via homologous recombination to form two final barcode acceptor strains: SHA345 and SHA349. Random barcode library 1 (lox66-Barcode-3'URA3-KanMx) and random barcode library 2 (KanMX-5'URA3-Barcode-lox71) amplicons were generated by PCR amplification of two plasmid barcode libraries, pBAR4_L1 and pBAR5_L1 (Supplementary Note 1), and then integrated by homologous recombination into theYBR209W locus of SHA345 and SHA349, respectively. Transformants were picked and arrayed into 96-well plates, and the barcode sequence in each well was identified by Sanger sequencing. (B) Haploid PPiSeq library construction. (1) Haploid strains expressing PCA hybrid proteins of interest tagged with the C-terminal portion of mDHFR (Prey ORF-DHFR[3]-HygMX) were each mated with different SHA345 + BCs. Similarly, haploid strains expressing PCA hybrid proteins of interest tagged with the N-terminal portion of mDHFR (Bait ORF-DHFR[1,2]-NatMX) were each mated with different SHA349 + BCs. (2) Diploid cells were selected using the KanMX and HphMX or KanMX and NatMX selection markers. (3) Diploids were transferred to sporulation media. (4) Spores were transferred into media to select for haploids of the correct mating type that contain both a barcode and a split DHFR construct. Mating of these haploid strains and selection for double barcode libraries is detailed in Figure 1 in the main text.

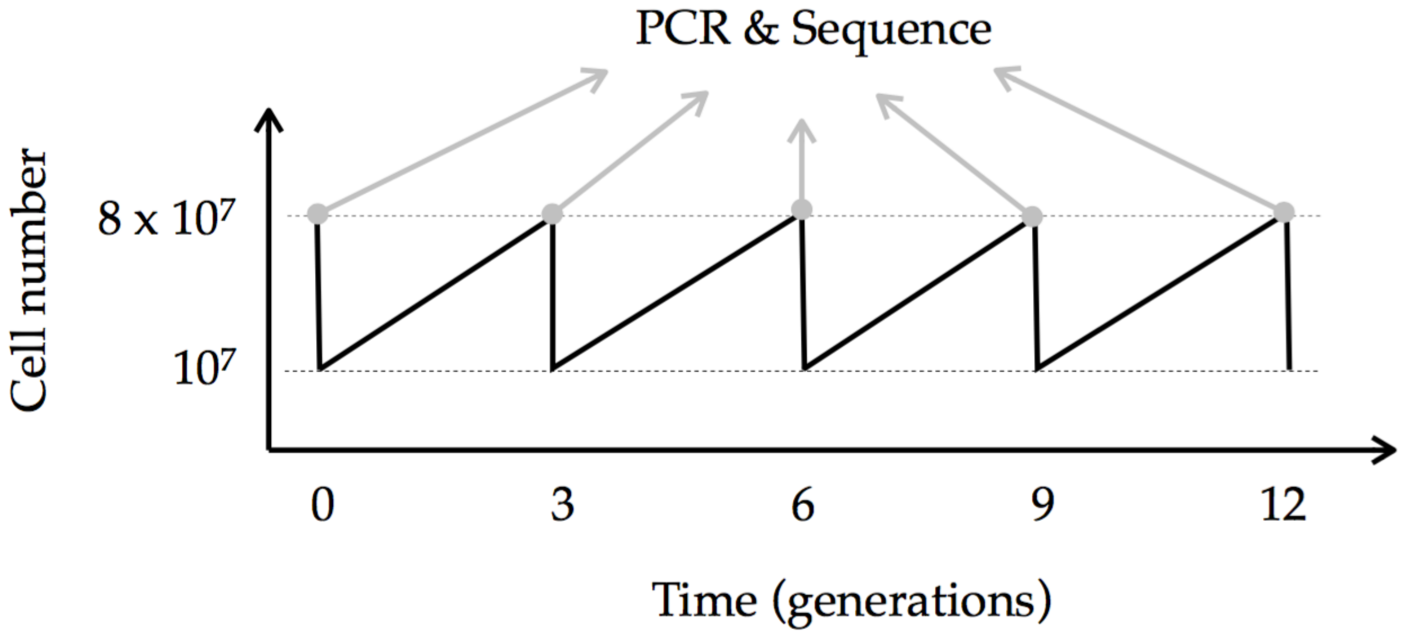

Supplementary Figure 2. Schematic of the pooled competition assay. Cells are passaged through multiple growth bottleneck cycles. At each passage cells are harvested for sequencing which enables a census of the population to be taken and the relative frequencies of the genotypes to be determined.

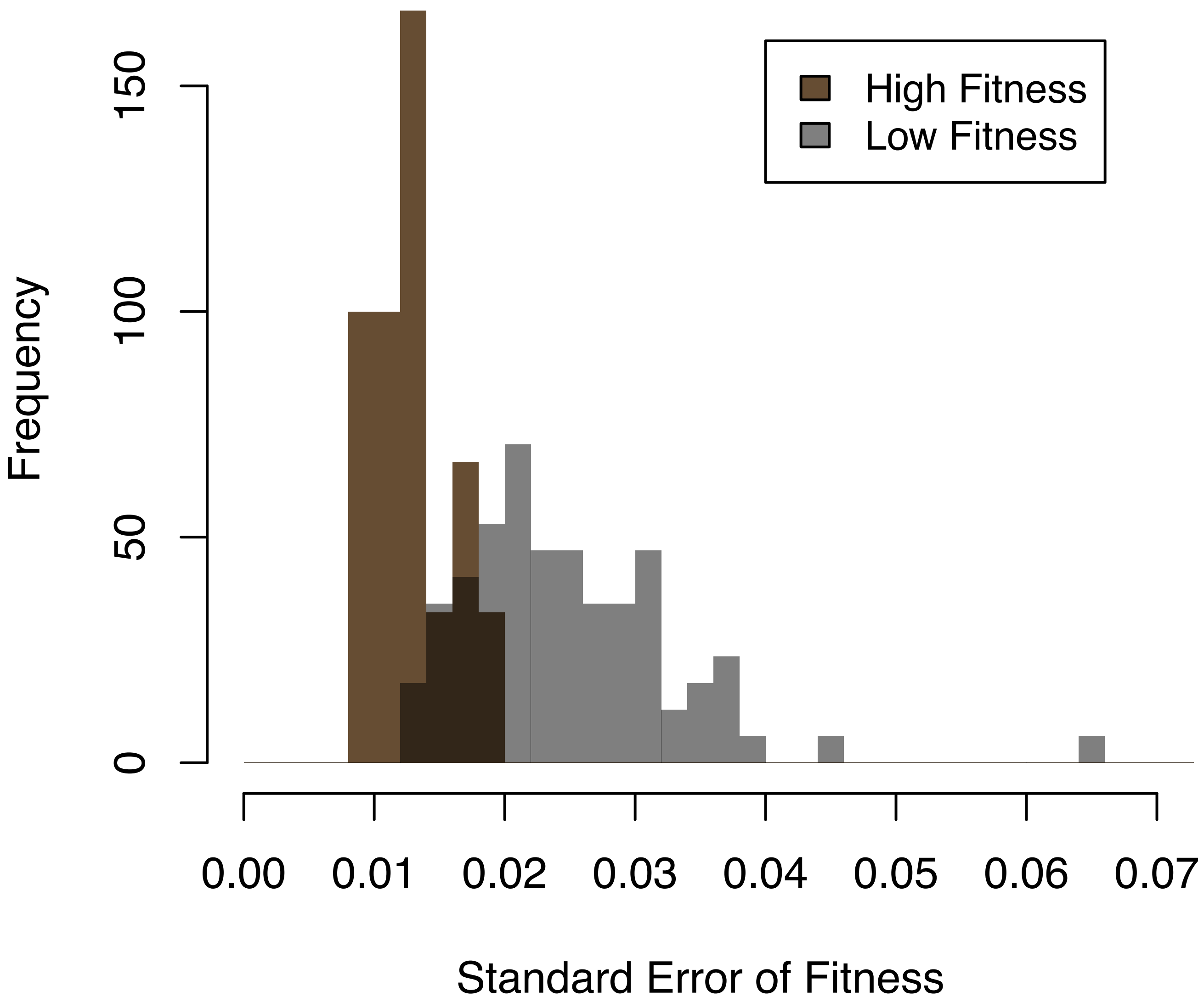

Supplementary Figure 3. Histograms of the standard error of fitness estimates of high fitness (brown, $x > 0.07$) and low fitness (grey, $x \leq 0.07$) PPiSeq strains.

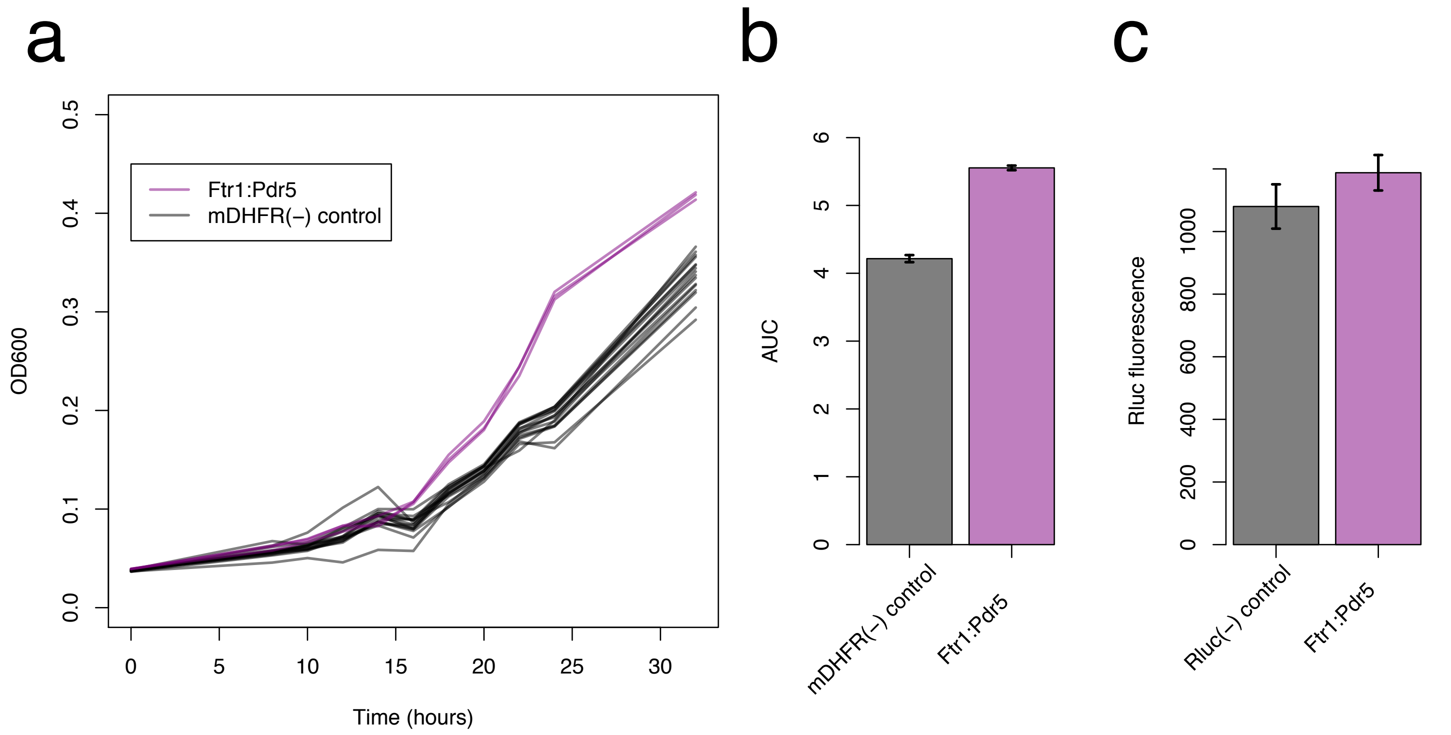

Supplementary Figure 4. Validation Ftr1:Pdr5 PPI. (A) The OD600 trajectories of the Ftr1-F[1,2]:Pdr5-F[3] split mDHFR PCA strain (purple) and a strain that lacks mDHFR fragments (grey). (B) Barplot of the Area Under the Curve (AUC) for strains in (A). Error bars are SEM, $p = 2 \times 10^{-11}$, Student's t-test. (C) Barplot of the Ftr1:Pdr5 split Renilla luciferase (Rluc) strain (purple) and a control that lacks any Rluc fragments (grey). Error bars are SEM, p = 0.25, Student's t-test.

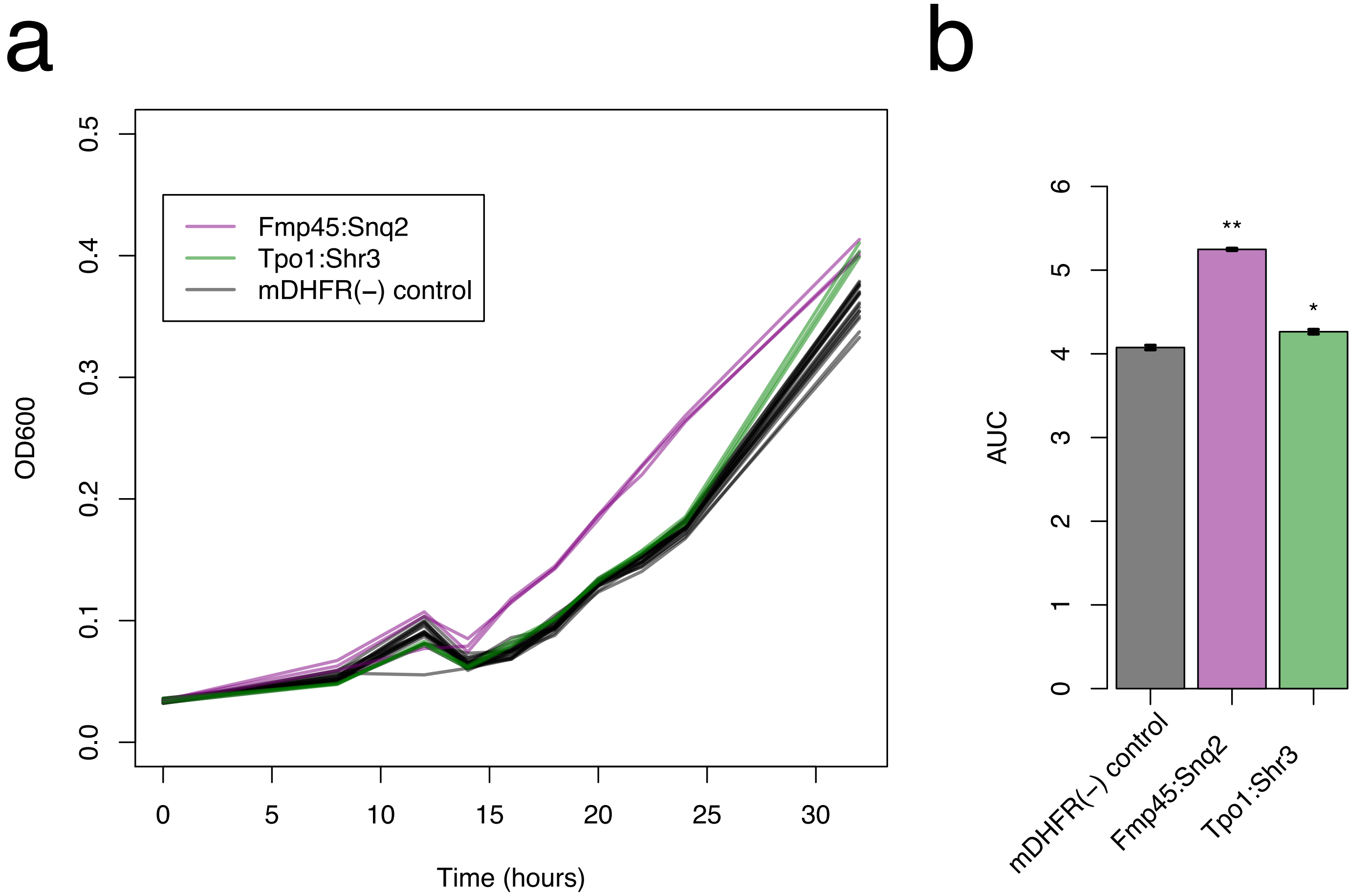

Supplementary Figure 5. Validation of false negatives. (A) The OD600 trajectories of split mDHFR PCA strains Fmp45-F[1,2]:Snq2-F[3] (purple) and Tpo1-F[1,2]:Shr3-F[3] (green), and a strain that lacks mDHFR fragments (grey). (B) Barplot of the Area Under the Curve (AUC) for strains in (A). Error bars are SEM, * $p < 0.01$, ** $p < 10^{-15}$, Student's t-test.

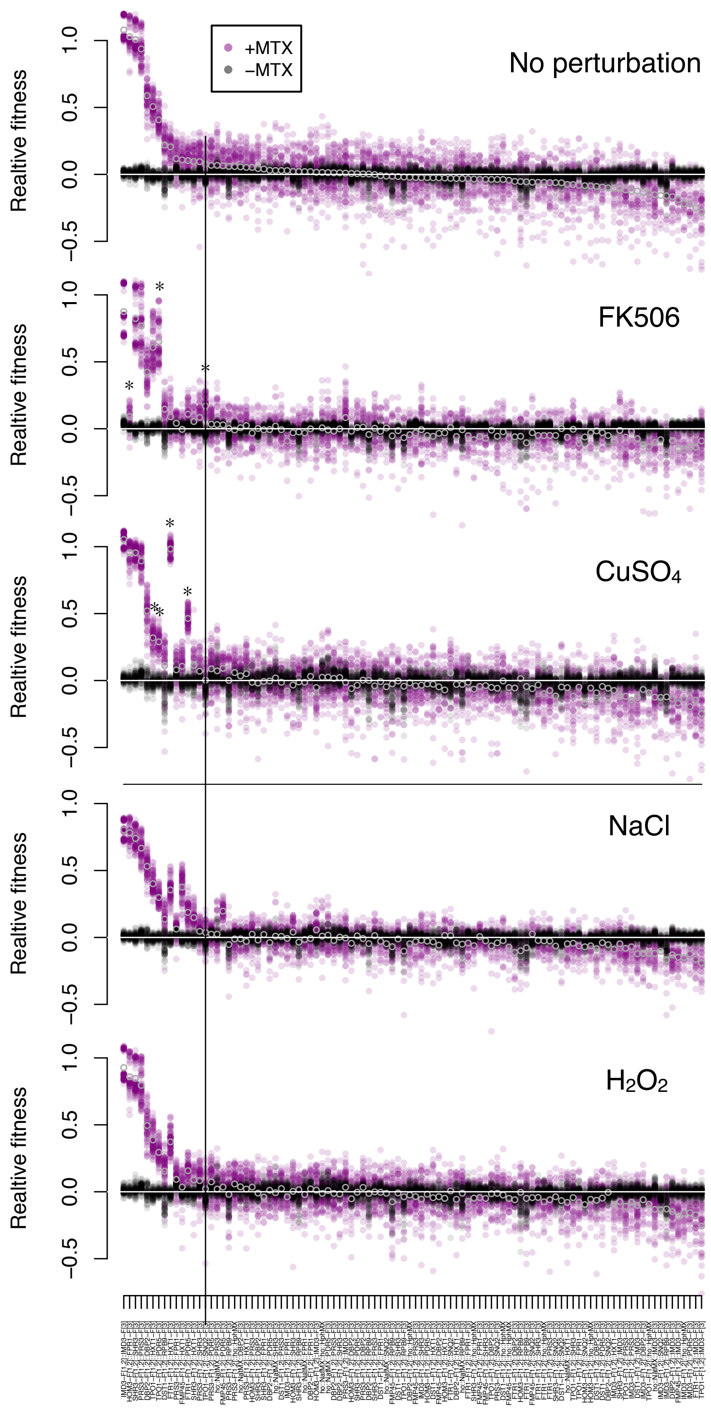

<u>Supplementary Figure 6.</u> Relative fitnesses of protein fragment pairs grown in five environments in the absence (black) or presence (purple) of MTX. Each protein fragment pair is assayed with 25 unique double barcodes across 3 growth replicates for a total of ~75 fitness estimates (PPI score). Hollow grey circles indicate the mean fitness of the protein fragment pair in MTX across all measures. PPIs are ranked according to their fitness in the benign environment (no perturbation) and rankings are maintained between plots.

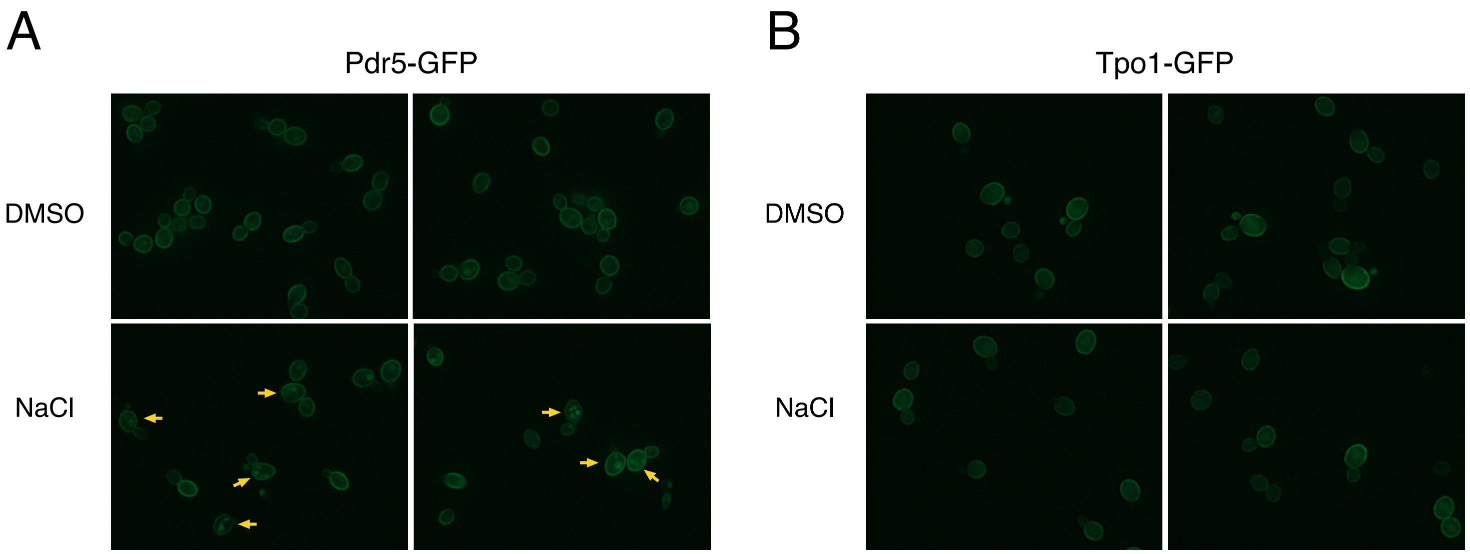

Supplementary Figure 7. Micrographs of diploids containing one copy of Pdr5-GFP (A) and Tpo1-GFP (B) grown in media containing DMSO (0.5%) or NaCl (175 mM). In DMSO, both constructs localize to the plasma membrane. Arrows show the internalization of Pdr5-GFP in NaCl.

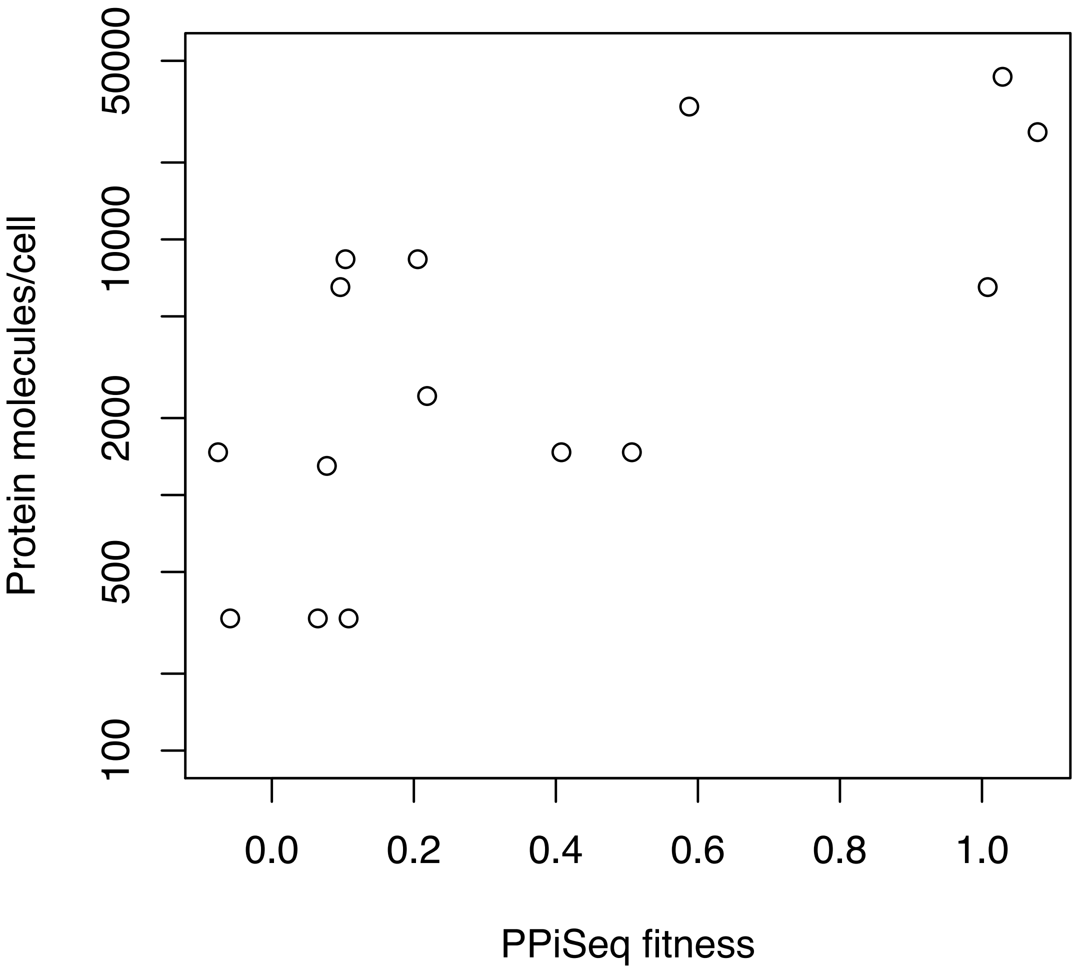

Supplementary Figure 8. PPiSeq fitness correlates with protein abundance. The fitness of PPIs detected in this study or elsewhere plotted against the abundance[1] of the least abundant protein in each PPI pair. Spearman's rho = 0.68.

Supplementary Note 1: PPiSeq strain construction

Construction of plasmid backbones.

pBAR1 has been described[2]. pBAR4 and pBAR5 were cloned from the following sources (all available from EUROSCARF) by standard methods: 1) plasmid backbone / bacterial origin from pAG32, 2) kanMX[3] from pUG6, 3) Gal-Cre from pSH63, 4) URA3 from pSH47, 5) artificial intron, random barcodes and loxP sites were synthesized de novo (IDT).

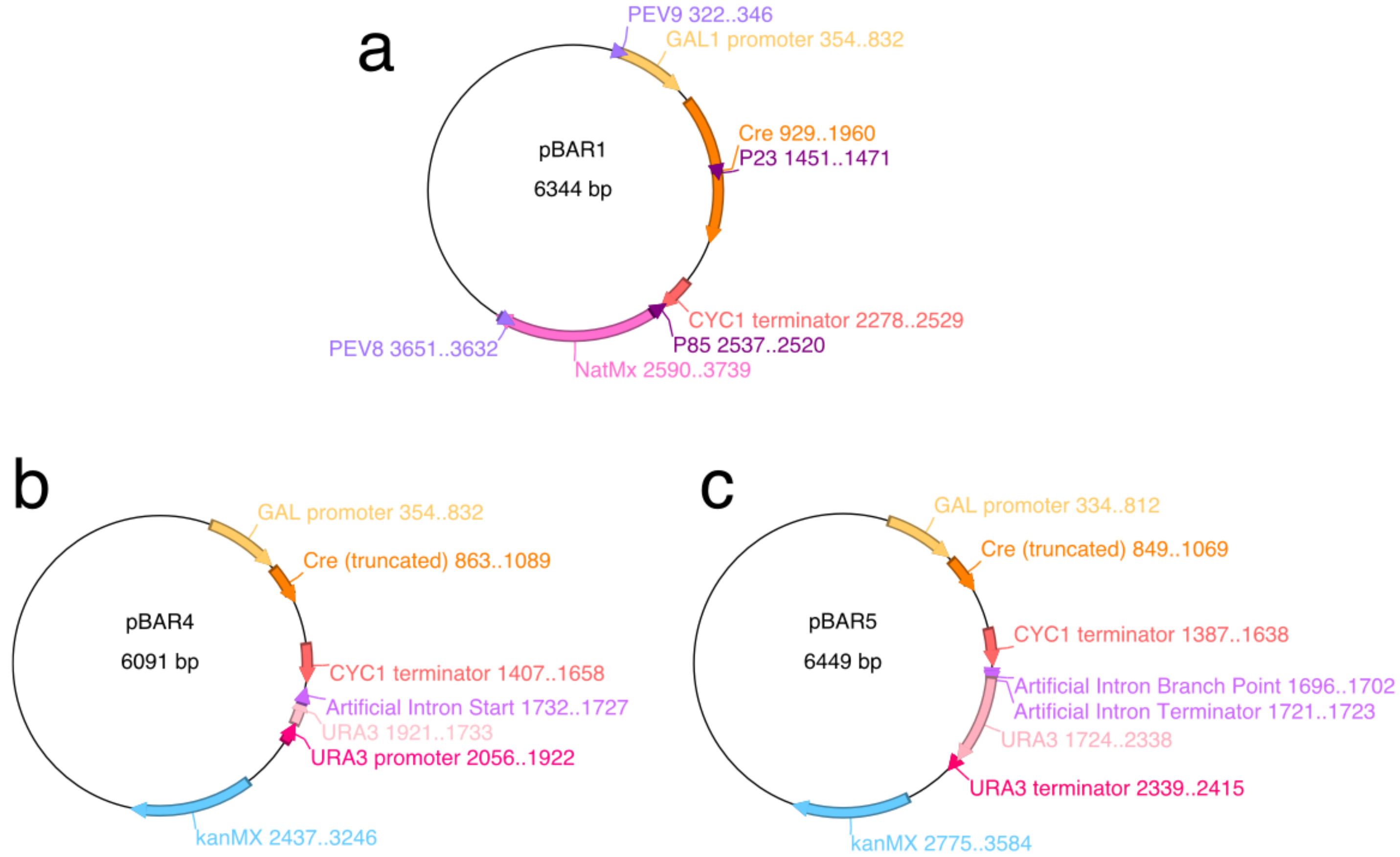

Construction of plasmid random barcode libraries.

Random barcodes were inserted into pBAR4 and pBAR5 by ligation. Primers containing a KpnI restriction site, a random 20 nucleotides, lox71 or lox66 [4,5] sites, and a region of homology to the plasmids were ordered from IDT using the “hand mixed” option:

P84 (lox66) =
CCAGCTGGTACCNNNNNAANNNNNTTNNNNNTTNNNNNATAACTTCGTATAGCATACATTATACGAACGGTAGGCGCGCCGGCCGCAAAT

P85 (lox71) =
CCAGCTGGTACCNNNNNAANNNNNTTNNNNNTTNNNNNATAACTTCGTATAGCATACATTATACGAACGGTAGGCGCGCCGGCCGCAAAT

Random sequences were limited to 5 nucleotide stretches to prevent the inadvertent generation of restriction sites. To construct the pBAR4 plasmid library, P85 and and P23 (GCCGAAATTGCCAGGATCAGG) primers were used to amplify a portion of pBAR1. Both the PCR product and pBAR4 were cut with KpnI and XhoI restriction sites and ligated together to generate plasmids containing a lox71 site and a random barcode. Ligation products were inserted into DH10B cells (Life Technologies) by electroporation, allowed to recover from electroporation in liquid media for 30 minutes, and plated onto 12 LB-Ampicillin plates at a density of ~6000 CFU/plate, a total of ~72,000 colonies. During the recovery period in liquid media, some fraction of the

cells could have undergone a cell cycle, meaning that our true library complexity is likely to be less than the number of colonies we observe. Colonies were pooled in 900 ml LB-Ampicillin and a fraction of the pool was used directly for plasmid preps to generate the plasmid library (pBAR4-L1) (Supplementary Fig. 9A). Similar methods were used with P84 (lox66) and pBAR5 to construct pBAR5-L1, a library containing ~120,000 barcodes (Supplementary Fig. 9B). The final barcoded plasmid libraries are pBAR4_L1 and pBAR5_L1. pBAR4_L1 contains a partially crippled loxP site (lox66) [4,5], the barcode region, the 3' end of URA3 gene preceded by part of an artificial intron and the KanMX dominant drug resistant marker[6]. pBAR5_L1 contains a complementary partially crippled loxP site (lox71) [4,5], the barcode region, the 5' end of URA3 gene followed by part of an artificial intron[6], and the *KanMX* dominant drug resistant marker[3].

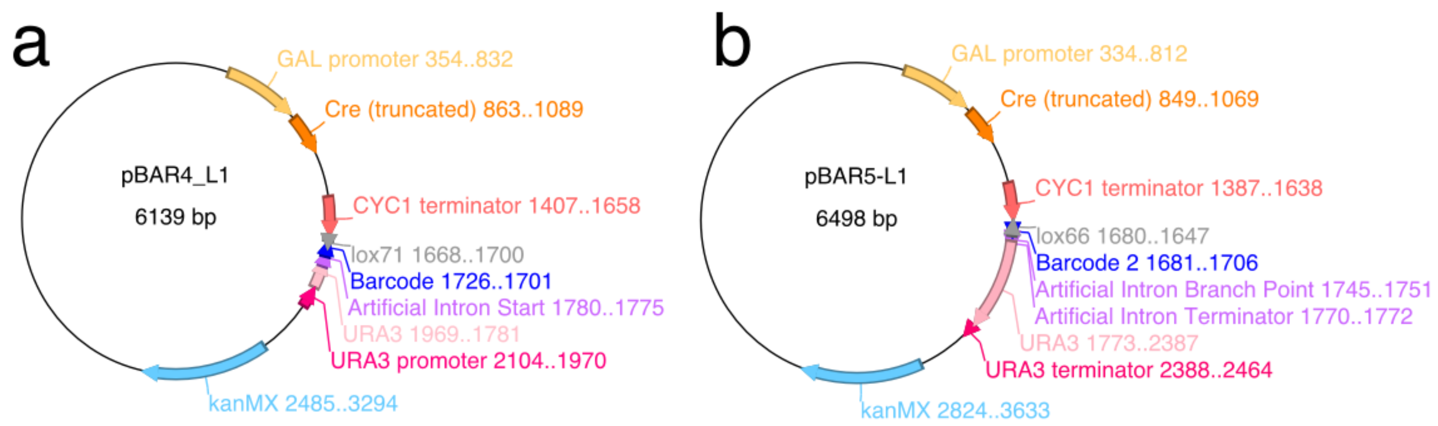

Construction of barcode acceptor strains.

Barcode acceptor strains are derived from BY4741 (*MATa, his3Δ1, leu2Δ0, met15Δ0, ura3Δ0*) and BY4742 (*MAT∝; his3Δ1; leu2Δ0; lys2Δ0; ura3Δ0*) [7]. First, Gal-Cre and NatMX was inserted the the YBR209W locus in opposite orientations via homologous recombination. Disruption of YBR209W has been previously demonstrated to have no impact on fitness. [8]. For the BY4741 insertion, pBAR1 sequence was amplified with the following primers:

P102 = GCTTGCGCTAACTGCGAACAGAGTGCCCTATGAAATAGGGGAATGCGCACTTAACTTCGCATCTG

P103 = GTTCTTTGCTTTTTTTCCCCAACGACGTCGAACACATTAGTCCTACATATCATACGTAATGCTCAACCTT

Underlined sequences correspond to sequences flanking the dubious open reading frame, YBR209W. The PCR product, containing Gal-Cre and the NatMX selectable marker, was inserted into the genome by homologous recombination [9]. For BY4742, Gal-Cre-NatMX was placed in the opposite orientation using the following primers:

PEV8 = GTTCTTTGCTTTTTTTCCCCAACGACGTCGAACACATTAGTCCTACGCACTTAACTTCGCATCTG

PEV9 = GCTTGCGCTAACTGCGAACAGAGTGCCCTATGAAATAGGGGAATGCATATCATACGTAATGCTCAACCTT

Second, we PCR amplified the dual magic marker (*MFapr1-HIS3-MF∝1pr-LEU2*) from strain UCC8600 [10-12], and inserted it at the *CAN1* locus in both the BY4741 and BY4742 derivative. The promoters *MFa1pr* and *MF∝1pr* are only active in *MATa* and *MAT∝* haploids, respectively. Populations of *CAN1/can1:: MFApr1-HIS3-MF∝1pr-LEU2* diploids can be easily converted to either *MATa* or *MAT∝* haploids by growing on media containing canavanine (for selection against diploids) but lacking histidine or leucine, respectively. Final barcode acceptor strains are SHA345 (*MATa, his3Δ1, leu2Δ0, met15Δ0, ura3Δ0 ybr209w::(F)GalCre-NatMX, can1::MFApr1-HIS3-MF∝1pr-LEU2*)
and SHA349 (MAT∝, his3Δ1, leu2Δ0, lys2Δ0, ura3Δ0, ybr209w::(R)GalCre-NatMX can1::MFApr1-HIS3-MF∝1pr-LEU2), where F and R represent opposite orientations relative to the centromere.

Construction of yeast random barcode libraries.

The barcode region of pBAR4_L1 and pBAR5_L1 were PCR amplified with P40, and PEV8 and PEV9, respectively.

P40 = CAACCTGAAGTCTAGGTCCTATT

PCR products from pBAR4_L1 (containing *lox66-Barcode-3'URA3-KanMX)* and pBAR5_L1 (containing *lox71-Barcode-5'URA3-KanMX)* were integrated by homologous recombination into SHA345 and SHA349, respectively, replacing the NatMX marker to yield SHA345+BC (*MATa, his3Δ, leu2Δ, met15Δ, ura3Δ, ybr209w::GalCre-lox66-Barcode-3'URA3-KanMX, can1:: MFa1pr-HIS3-MF∝1pr-LEU2*) and SHA349+BC (*MAT∝, his3Δ, leu2Δ, lys2Δ, ura3Δ, ybr209w::KanMX-5'URA3 -Barcode-lox71-GalCre, can1:: MFa1pr-HIS3-MF∝1pr-LEU2*). Transformants were picked and arrayed into 96-well plates for storage and further characterization. Each SHA345+BC and SHA349+BC strain was assayed for growth on YDP +kanamycin (for *KanMX*), YPD + nourseothricin (for loss of *NatMX*). Additionally, each strain was mated to a complementary tester strain, and plated on CM +galactose -uracil to test for a functional *barcode-loxP-1/2URA3* construct. Barcoded strains that passed quality, we next Sanger sequenced at the barcode locus to identify the random barcode sequence. Strains that contain the same barcode were removed from the plate arrays. To check for errors in the library, we next employed an arrayed mating strategy whereby arrayed SHA345+BC plates were pairwise mated to arrayed SHA349+BC plates. Arrayed matings were plated CM +galactose -uracil to select for diploids that have undergone Cre-lox recombination to generate double barcodes. The diploids were pooled, double barcodes from these pools were PCR amplified with a plate specific primer pair, and multiple plate matings were sequenced together on an Illumina MiSeq (see below). Unexpected double barcode reads (which indicate that there was an error in Sanger sequencing or arraying, or a well contained a mix of multiple barcodes) was used to prune the barcode libraries. In total, we generated a verified library 1137 *MATa* SHA345+BC and 844 *MATα* haploid barcode strains.

Haploid PPiSeq library construction.

Nine haploid strains expressing PCA hybrid proteins of interest tagged with the N-terminal portion of mDHFR (HOM3-F[1,2]-NatMX, DST1-F[1,2]-NatMX, TPO1-F[1,2]-NatMX, FMP45-F[1,2]-NatMX, FTR1-F[1,2]-NatMX, IMD3-F[1,2]-NatMX, DBP2-F[1,2]-NatMX, SHR3-F[1,2]-NatMX, PRS3-F[1,2]-NatMX) and one negative control strain (ho::NatMX) were each mated with five different SHA349+BC strains. Similarly, nine haploid strains expressing PCA hybrid proteins of interest tagged with the C-terminal portion of mDHFR (FPR1-F[3]-HphMX, RPB9-F[3]-HphMX, SNQ2-F[3]-HphMX, PDR5-F[3]-HphMX, HXT1-F[3]-HphMX, IMD3-F[3]-HphMX, DBP2-F[3]-HphMX, SHR3-F[3]-HphMX, PRS3-F[3]-HphMX) and one negative control strain (ho::HphMX) were each mated with five different SHA345+BC strains. The haploid PCA strains were described in [13] and are commercially available at Dharmacon. Diploids were selected on YPD + G418 + nourseothricin or YPD + G418 + hygromycin B, respectively. The resulting diploids (i.e. two sets of 50 strains) were then sporulated by growing them overnight in YPD to saturation in 96-well microtiter plates at 100 µl per culture, and on the following day washing the pellets twice with water and resuspending the pellets in 'enriched sporulation media' [14]. The sporulation cultures were incubated in 96-well microtiter plates at 24°C with continuous shaking at 200 rpm. Spore counts were about 10 - 20% after one week. 10 µl of every culture was then transferred into 5 ml of YNB + ammonium sulfate + dextrose + leucine + uracil + canavanine + G418 + nourseothricin to select for MATa haploids with a barcode, GENE-F[1,2]::NatMX (and MET+, LYS+) or YNB + ammonium sulfate + dextrose + histidine + uracil + canavanine + G418 + hygromycin B to select for MATalpha haploids with a barcode, GENE-F[3]::HphMX (and MET+, LYS+) and grown for 3 days to saturation.

Supplementary Note 2: Correction for putative PCR chimeras

Most double barcode lineages are expected to be near extinction by 12 generations of growth (**Fig. 2a**). We plotted the total number of reads for each double barcode (y-axis) against the total number of reads for each barcode 1 (BC1) multiplied by the total reads of barcode 2 (BC2, x-axis) across all conditions after 12 generations of competitive pooled growth. BC1 and BC2 frequencies are calculated by ignoring the other half of the double barcode.

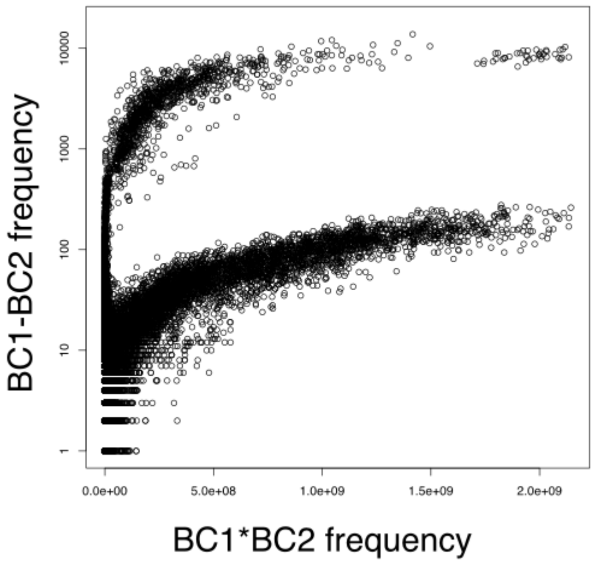

This plot revealed a significant fraction of unexpected double barcodes remained (lower band). These unexpected double barcodes are generally confined to barcode pairs where both barcodes are abundant in the pool for other reasons. That is, they participate in a PPI (upper band), only with a different barcode partner. The most parsimonious explanation is that these double barcodes are not truly in the template pool, but rather are technical errors that result from PCR chimeras: two barcodes that stem two different templates that are merged during PCR. To remove these artifacts, we replotted this relationship except the y-axis is linear and only the lower band is plotted at BC1*BC2 frequencies greater than 10^8.

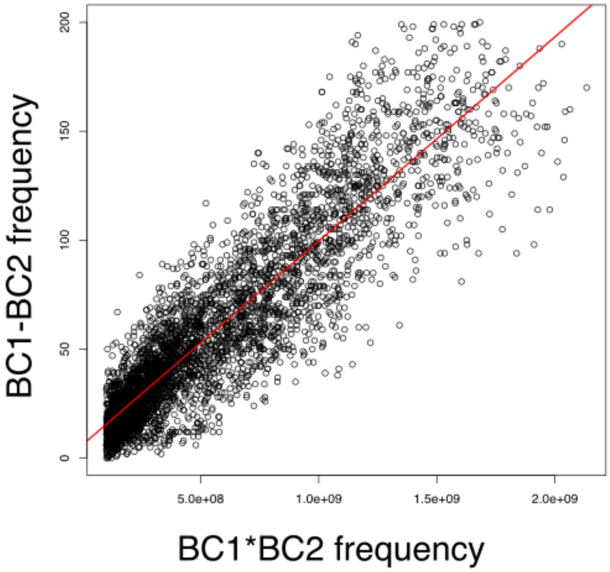

The linear fit (red line) shows that there is a strong linear correlation between the number double barcode reads in this class and the product of the number of reads for each barcode half irrespective of its barcode partner (slope = 9.36×10^{-8}, intercept = 6.14, Pearson's r = 0.903). We therefore used used this fit to correct all double barcode reads for PCR chimeras.

Supplementary Note 3: Fitness estimation by lineage tracking

We use the corrected double barcode reads at 0, 3, 6, 9, and 12 generations to estimate the fitness of each double barcode PPiSeq strain in each condition and replicate. In competition assays, the "fitness" is defined as a relative growth rate: the relative increase in frequency per unit time of one genotype over another. Here, we measure relative to a "null" strain with no PCA constructs (ho::NatMX/ho::HphMX), whose fitness is then defined to be $x = 0$. Using the frequency of each double barcode to infer the fitness, x, of each lineage (between time points t and $t + \delta t$) relative to this null strain is then straightforward:

$$x = \bar{x}(t) + \frac{\ln\left(f(t+\delta t)\right) - \ln\left(f(t)\right)}{\delta t} \tag{1}$$

where $\bar{x}$ is the mean fitness of the population, defined as

$$\bar{x}(t) = \sum_{\text{lineages } i} x_i f_i. \tag{2}$$

Because of the differences in fitness between strains, the mean fitness can change substantially over short periods of time, even at the very beginning of the assay. Accurate inferences of fitness from frequency data must take this changing mean fitness into account.

Linear regressions can have high errors of fitness. The simplest way of estimating the relative fitnesses would be to perform a linear regression on the (log) relative frequencies. However in most situations, a linear regression performs poorly because, as the mean fitness of the population increases, trajectories begin to curve and linear regression will no longer accurately capture the true relative growth rates (Figure 2A, main text). Sometimes, if the mean fitness does not increase significantly early on, restricting analysis to the first two time points allows linear regression to perform reasonably well. However, the rate at which the mean fitness changes depends strongly on the pool of genotypes being tested and the environment in which they are grown, so this method can not be generalized. Additionally, subtle fitness differences will often go undetected when restricted to just two time points because the noise around any one time may be high. Incorporation of additional time points (when the mean fitness is changing) therefore has the potential to significantly decrease fitness estimate errors.

A maximum likelihood method to reduce fitness errors. To improve fitness estimates over linear regression, we use a maximum likelihood algorithm to infer relative fitnesses. Our algorithm maximizes:

$$\text{Probability}(\ \texttt{relative frequency data} \mid \texttt{fitness estimates \& initial frequency estimates}\ ) \tag{3}$$

The advantage of such an approach is that it makes use of all the data. As we show in the comparisons to simulated data sets this approach can significantly improve fitness estimates: reducing the errors on high fitness genotypes by an order-of-magnitude under conditions similar to our experiment. Improvements of our likelihood maximization process over a linear fit will, of course, depend on the environment, the pool of genotypes being tested, and the sampling frequency.

Interactions though the mean fitness. One key subtlety in performing any optimization to determine the "best" fitness estimates is that one cannot optimize each lineage independently. A change in the estimate for the fitness of lineage 1, say, impacts the likelihoods of all other lineages, particularly if lineage 1 is very fit. We discuss this subtlety in steps 10–12 of the algorithm below in reference to how best to update guesses to search for the maximum likelihood position.

What functional form should be chosen for the likelihood function? In general there are a number of stochastic processes that determine the relative frequency inferred from unique sequencing reads given an initial frequency and fitness. These include sampling at the sequencer (i.e. finite read depth), PCR amplification noise and noise inherent to the growth process of the cells and sampling at bottlenecks

("genetic drift"). Details of the contribution of each of these effects have been outlined in previous work[2,15]. In the data considered here the population size ($N \approx 10^7$) is far larger than the read depth at a typical time point ($D \approx 5 \times 10^5$). Therefore sampling at the sequencer dominates the noise with genetic drift adding a very minor correction to this (see below "Errors on frequency"). We therefore assume changes in relative frequency from time point to time point are deterministic, with all noise introduced at the sequencing stage. Extending our algorithm to include other forms of noise would be straightforward. In previous work[2] we have found that:

$$\ln P(r|f) \approx \frac{1}{2}\ln\left(\frac{(Df)^{1/2}}{4\pi\kappa r^{3/2}}\right) - \frac{\left(\sqrt{r}-\sqrt{Df}\right)^2}{\kappa} \tag{4}$$

is an accurate functional form for the noise, so we use this in our likelihood estimates. Here κ is a (free) noise parameter $O(1)$ that can be fit from the data. Of particular importance is that this form has an exponential rather than Gaussian tail.

Algorithm

1. Start by making an initial guess at the initial frequencies $\boldsymbol{f}$ and fitnesses $\boldsymbol{x}$ for all lineages (these are vectors whose entires are the values for the first, second lineage etc... down to the 2,500th lineage). A good guess at the initial frequencies comes from looking at the relative frequency of the lineages at t_0:

$$f_i = \frac{r_i(t_0)}{D(t_0)} \tag{5}$$

where r_i is the number of reads on the ith lineage and D the read depth (both at $t = 0$). A reasonable first guess for the fitnesses comes from performing a linear regression on the log-transformed trajectories:

$$x_i = \frac{\ln\left(f_i(t+\Delta t)\right) - \ln\left(f_i(t)\right)}{\Delta t} \tag{6}$$

2. Given these initial guesses we want to calculate the likelihood of the data under the assumption that competition between lineages is only via the mean fitness and that no lineages accumulate any additional beneficial mutations, so that fitnesses remain constant in time.

3. Use the fitnesses $\boldsymbol{x}$ and initial frequencies $\boldsymbol{f}(t_0)$ to estimate the initial mean fitness $\bar{x}(t_0)$

$$\bar{x}(t_0) = \boldsymbol{x} \cdot \boldsymbol{f}(t_0) \tag{7}$$

4. Use the fitness x_i and the initial mean fitness $\bar{x}(t_0)$ to predict the frequencies at the next time point:

$$f_i(t_0 + \Delta t) = f_i(t_0)\exp\left[(x_i - \bar{x}(t_0))\Delta t\right] \tag{8}$$

5. Recalculate the new mean fitness at this later time point:

$$\bar{x}(t_0 + \Delta t) = \boldsymbol{x} \cdot \boldsymbol{f}(t_0 + \Delta t) \tag{9}$$

6. Iterate this procedure until the frequencies of all lineages at all time points are predicted (as well as mean fitness trajectory):

$$\{\boldsymbol{f}(t_0), \boldsymbol{f}(t_1)...\boldsymbol{f}(t_k)\} \qquad \text{and} \qquad \bar{x}(t) \tag{10}$$

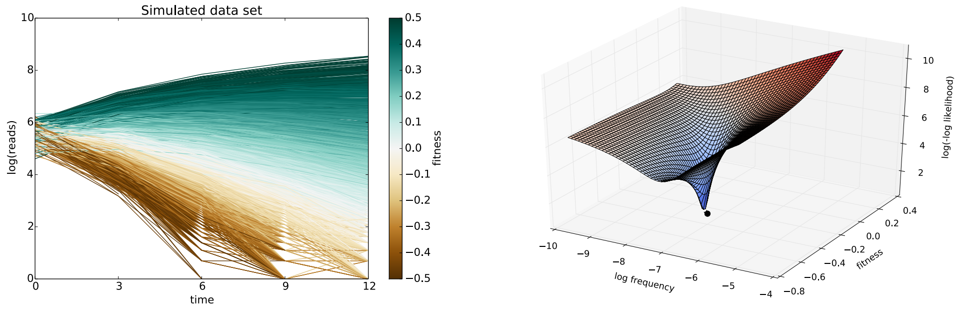

a **b**

7. The (log) probability distribution across reads, r, given some read depth, D, and true frequency, f, of the lineage is calculated using

$$\ln P(r|f) \approx \frac{1}{2}\ln\left(\frac{(Df)^{1/2}}{4\pi\kappa r^{3/2}}\right) - \frac{\left(\sqrt{r}-\sqrt{Df}\right)^2}{\kappa} \tag{11}$$

where κ is the noise parameter whose magnitude is on the order of 1 (typically between 1 and 3) and can be obtained by fitting.

8. The log likelihood of the data given the model is then obtained by summing over all time points. The total likelihood L of all data given the guesses across all lineages is then obtained by summing across all lineages. This value L is a function of $\boldsymbol{x}$ and $\boldsymbol{f}(t_0)$, which are our "guesses".

$$L(\boldsymbol{x}, \boldsymbol{f}(t_0)) \tag{12}$$

9. The aim is to maximize this likelihood by making small changes to our guesses and accepting those that increase the likelihood. However, because of the interaction through the mean fitness, it is extremely inefficient to make *random* steps away from the current guess and re-evaluate the likelihood each time as some optimization algorithms would implement. The inefficiency comes from the fact that any change to any fitness requires re-calculating the likelihood for all other lineages because of the interaction through the mean fitness.

10. Instead, we implement a "smart" guess by realizing that the interaction through the mean fitness is rather weak. What this means in practice is that maximizing the likelihood of each lineage independently, assuming that the mean fitness does not change, should be a good approximation to the true maximum likelihood guess and hence should be a sensible next guess. We therefore choose this the way of updating our guesses for frequency and fitness.

11. Once this new guess is made, the trajectories are calculated in a way that is self-consistent with the predicted mean fitness as outlined in steps 3–6. If the guess increases the likelihood, it is accepted.

12. This process is repeated until the algorithm converges (no steps can increase the likelihood further).

13. The final guesses for the frequency and fitness vectors are then assigned to me the maximum likelihood guesses.

14. This algorithm is *not* guaranteed to converge to the global maximum since it is deterministic rather than stochastic. However, by examining a large number of likelihood surfaces (as shown in panel **b** the figure below) we found no cases where the algorithm was trapped in a local maximum (because landscapes are smooth). We verified this with simulations discussed below.

Applying the maximum likelihood algorithm above to a simulated data set with 2500 lineages (panel **a** of the figure above) results in accurate inferences of the fitness (see figure below). The algorithm improves upon linear regression substantially, particularly for lineages with positive fitness. Lineages with ($x > 0$) typically are measured across all 5 time points. Here the fact our algorithm uses all the data is important: it reduces the errors in fitness by an order of magnitude (from ± 0.1 down to ± 0.01). For lineages with negative fitness the improvement is more modest. Lineages with low fitness are typically pushed to low frequencies rapidly and the first two time points are therefore the most informative. It is therefore hard to improve substantially on the linear regression method which itself uses only the first two time points. We observe however that this is some improvement for lineages with moderately negative fitness $-0.3 < x < 0$. Here fitness errors come down by about a factor of two (from ± 0.1 to about ± 0.05)

Comparison to simulated data set. To verify that this algorithm does indeed work well and to quantify the improvement it affords over a simple linear regression we ran it on a simulated data set (see figure below). The simulated data set was closely modeled on the experimental set-up. Specifically:

1. Two vectors (of length 2,500) are created to serve as the true initial frequencies $\boldsymbol{F}$ and true fitnesses $\boldsymbol{X}$.

2. The initial frequencies $\boldsymbol{F}$ are drawn from a Gaussian distribution with mean $\mu = 1/2500 = 4 \times 10^{-4}$ and standard deviation $\sigma = 8 \times 10^{-5}$ with each entry being forced to be positive.

3. The fitnesses $\boldsymbol{X}$ are drawn from a distribution with density $\rho(x) = \exp(-|x|)$ where the range is restricted to being in the interval $-0.5 < X < 0.5$. This distribution means that most lineages have small fitness, while also ensuring there will also be lineages at the extremes of the range.

4. The frequencies of each lineage at subsequent time points are calculated via:

$$F_i(t+1) = F_i(t) \exp(X_i - \bar{X}(t)) + \eta \sqrt{\frac{F_i(t)}{N}} \tag{13}$$

   where the first term is the deterministic change in frequency due to fitness differences and the second term are stochastic changes due to genetic drift. $\bar{X}$ is the mean fitness $\boldsymbol{X} \cdot \boldsymbol{F}$ and η is a random variate from a Gaussian distribution with zero mean and unit variance which is used for the stochastic elements of genetic drift. Using this procedure frequency data is generated for each lineage out to 12 generations.

5. Every 3 generations we generate read counts by Poisson sampling the frequencies at a mean coverage of 200/lineage = $500,000$ total reads (typical of the data).

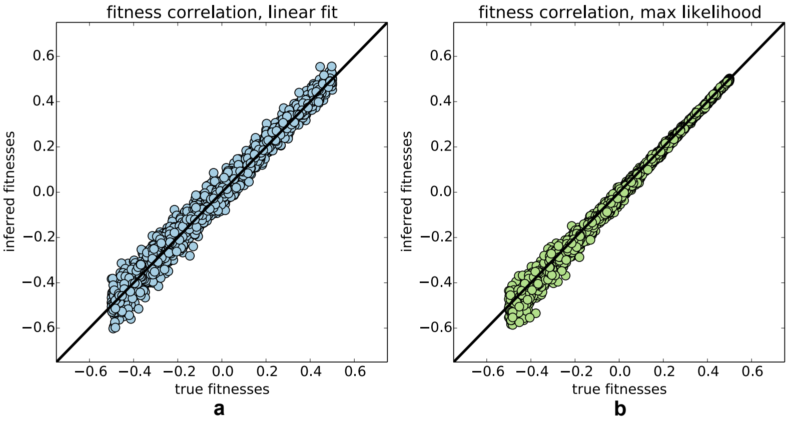

.

Robustness of fitness estimation to different distributions of strain fitness. The performance of the fitness inference is influenced by (i) the distribution of initial strain fitnesses in a given range, and, (ii) the absolute the range of initial fitnesses. To test the robustness of this fitness inference algorithm to these changes, we performed a further 4 simulations in which we altered both the distribution of strain fitness and the absolute range of fitness differences across strains.

Changing the distribution of initial strain fitnesses. The figure below shows simulations with relative fitness differences in the range $[0, 1]$ (fittest strain increases its frequency by a factor of $e \approx 2.72$ over the neutral per generation), similar to those observed in the experimental data. Panels (a) - (c) are for distributions of strain fitnesses with increasing skew to a few high fitness strains: (a) uniform (b) weighted as $\exp(-3 \times \text{fitness})$ and (c) weighted as $\exp(-6 \times \text{fitness})$. A small number of high fitness strains (c) actually improves fitness estimates as the mean fitness increases more slowly, enabling low fitness strains to remain at higher frequencies for longer (see narrowing of error in final column at low-fitness values). The general limits on this method can be explored by considering the worst case scenario (almost all the population has high fitness ($x_{min} + \Delta$)), then examining how well we can detect the fitness of a low fitness strain (x_{min}) initially present in n_0 cells. In the T generations before the next sample, the low fitness strain will decrease in abundance by a factor $\exp(-\Delta T)$. In order to make an estimate of its fitness it must at least be present at the following time point and so a general measurement limitation is:

$$n_0 \exp(-\Delta T) > 1 \tag{14}$$

Measuring very low fitness strains requires either finer time resolution (smaller T), a smaller fitness deficit relative to high fitness strains (smaller Δ) or for them to be spiked in at larger initial abundance (larger n_0). This result assumes that the sequencing depth is not limiting. In cases, where depth rather than cell number is the limiting factor in frequencies, then the same general result holds except r_0 (initial read number) replaces initial cell number (n_0),

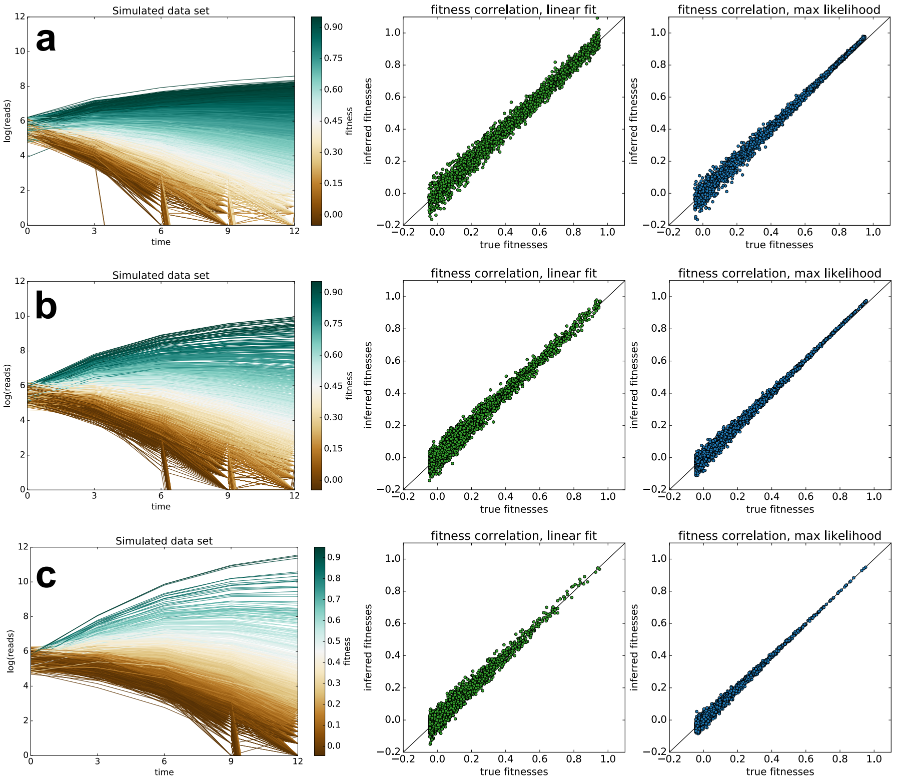

.

Changing the range of initial strain fitnesses. From the pervious discussion, for a given sampling time T and initial abundance n_0, the fitness difference Δ between the highest fitness and lowest fitness strains is important in setting the errors on the low fitness strains. To test this, and the criterion above, we tested how well the algorithm performed on two further simulated data sets with increasing Δ: (a, $\Delta \approx 1.0$), (b, $\Delta \approx 1.5$) and (c, $\Delta \approx 2.0$). As can be seen in the bottom row, for a $\Delta \approx 2.0$ and a $T = 3$, cells initially present in $n_0 \sim 10^3$ copies (the lower range of the numbers used in these simulations), the errors increase significantly on the lower fitness strains, and a small number are beginning to go extinct over the first time point. Beyond these values, accurate estimates for the fitness of the low-fitness strains becomes a major challenge and would have to be overcome by reducing Δ or T. We note however, that $\Delta > 1$ are not observed in our data and thus we not near this detection limit.

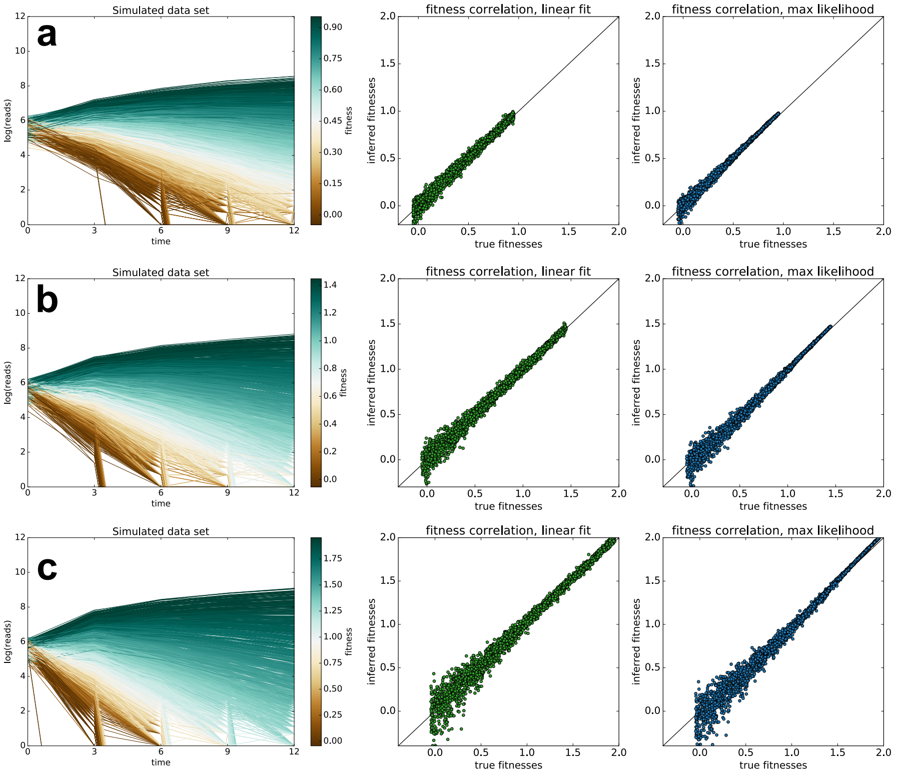

.

Supplementary Note 4: Analysis of errors

Errors on frequency measurement. The errors in frequency measurements for the vast majority of barcodes are characterized by counting noise i.e. noise where the variance is proportional to the mean. To validate this, we looked at frequencies of the same barcode measured across different replicates. If the noise is counting noise, then the standard deviation (i.e. typical error) in the frequency in replicate 1, say, should be:

$$\delta f_1 \sim \sqrt{\frac{f}{R_1}} \tag{15}$$

hence if we plot the magnitude of the difference in estimated frequencies between the two replicates divided by the mean frequency (the "coefficient of variantion") then

$$\frac{|f_1 - f_2|}{\bar{f}} \sim \frac{1}{\sqrt{f}} \left| \frac{1}{\sqrt{R_1}} - \frac{1}{\sqrt{R_2}} \right| \tag{16}$$

so counting noise behavior can be validated by checking that, as a function of the mean frequency, the coefficient of variation declines as $1/\sqrt{f}$. The constant of proportionality should be a small multiple of $1/\sqrt{R}$ where R is the sequencing depth. In the plot below we validate this by plotting the coefficient of variation in frequency between replicates as a function of mean frequency on log-log axes, on which a $1/\sqrt{f}$ scaling will have a gradient of $-1/2$. For barcodes at low frequency ($< 0.1\%$), their scaling broadly agrees with that predicted by counting noise with a coefficient between 1–3. The error in frequency of these barcodes is therefore dominated by the the noise that comes from finite read depth. Barcodes present at higher frequencies ($> 0.1\%$) begin to deviate from this scaling as has been previously pointed out[16]. Barcodes at higher frequency likely have non-negligible contributions from other noise processes such as PCR and DNA prep noise as well as a likely contribution from biological noise i.e. the comparisons between replicates in the above figure have been performed after 3 generations of independent growth. As discussed below, there are also sources of systematic errors which disproportionately affect high-frequency barcodes. We note however, that the errors associated with high-frequency barcodes are nonetheless generally much smaller than those of low frequency barcodes.

Systematic errors on fitnesses. To quantify the magnitude of systematic errors in fitness, we plot all correlations between fitness inferences across all replicates for each condition (correlation plots below). In most conditions we find a consistent story: high fitness barcodes in one of the three replicates typically demonstrate systematic differences in relative fitness with magnitudes up to ± 0.15. Interestingly these systematic effects only influence the high-fitness strains. Low fitness strains have no noticeable systematic effects (i.e. they are scattered symmetrically around the $x = y$). Systematic effects on pooled fitness measurements have been observed previously[16]. The most likely explanation for these systematic effects are due to estimations of the "mean fitness" over the last few time points. A slight underestimation of the mean fitness at late times, for example, would cause the estimates for all high fitness barcodes to be underestimated too. Such systematic effects influence the high-fitness barcodes more than the low

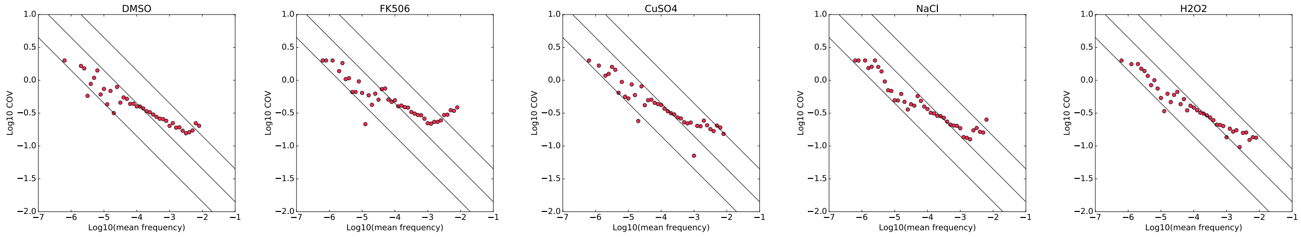

a **b** **c** **d** **e**

fitness ones because information from the later time points affects their estimates more (since they are at higher frequency at late times). Another plausible reason for these systematic effects is that the handful of high fitness strains that dominate the population at late times can modestly change the environment in which pooled growth is happening. This is consistent with the lack of systematic effects observed in previous pooled growth studies[2] which start with higher complexity pools and where no one strain increases enough to dominate the population. We hypothesize therefore that systematic effects will be reduced as the PPiSeq platform is scaled up. In this case, any one strain will constitute a small fraction of the pool and therefore makes it less plausible it can change the environment significantly.

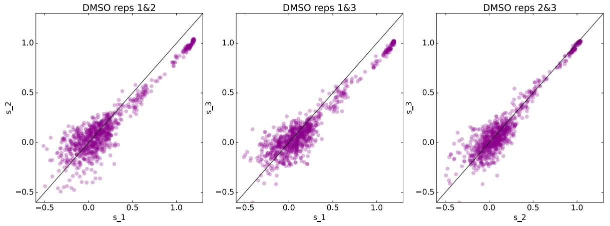

a

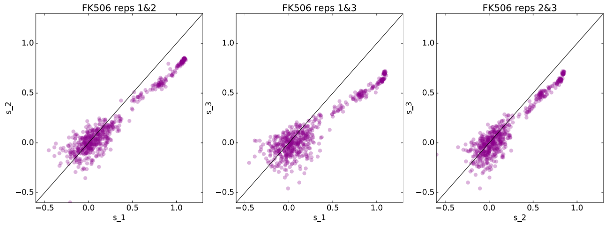

b

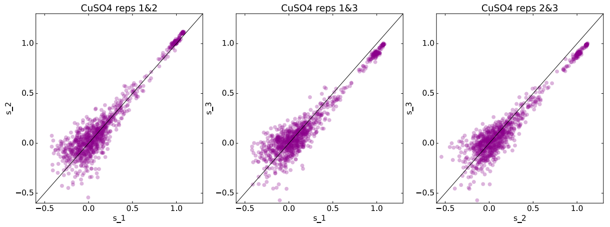

c

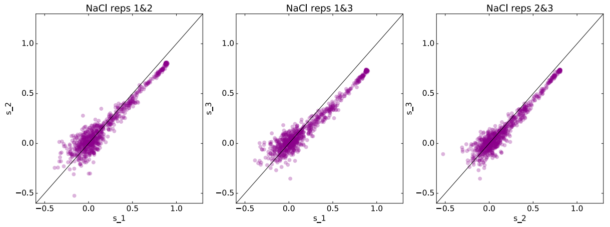

d

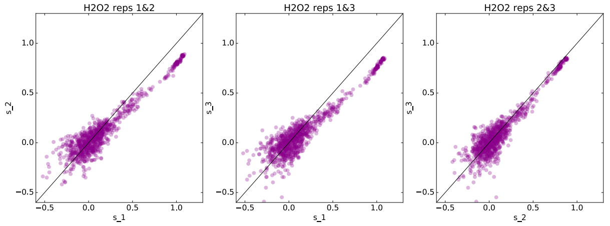

e

Supplementary Note 5: Microscopy of Pdr5-GFP and Tpo1-GFP

Haploid GFP tagged strains[17] Pdr5-GFP and Tpo1-GFP were streaked from frozen stocks onto YPD to recover isolated colonies. Each strain was mated to *MATα* ho::HphMx in YPD liquid media. After 12 h of mating, cells were plated onto SC-Met-Lys agar and grown for 48 h at 30 ºC to select for diploids. A fresh colony of each diploid was grown in 5 ml synthetic dextrose supplemented with standard concentrations of the amino acids histidine, leucine, and uracil for 24 h at 30 ºC, then diluted 1:11 into 5 ml of the same media supplemented DMSO (0.5%) and sodium chloride (175 mM). Cells were grown for and additional 54 h at 30 ºC, diluting 1:11 into fresh media every 24 h. Micrographs were collected on a Zeiss Axioplan2 microscope with a Zeiss mRM digital camera.

Supplementary Note 6: Mating and loxP recombination efficiency estimates

Mating efficiency estimation

A mating efficiency test between barcoded PCA strains was performed in quadruplicate. Barcoded *MATa* and *MATα* PCA pools were each grown in 50 ml YPD liquid media to saturation. The two pools were combined, and 1 x 10^{10} cells were plated onto a single YPD plate to mate. Cells were grown for 24 h at 30 ºC and the cell lawn was scraped into 10 ml of water. A cell count was taken to determine the total growth on the 2plate (~1.7-fold growth). Cells were spread onto plates YPD + CloNat + Hygromycin plates at densities of 1000, 2000, and 5000 cells/plate to estimate the number of diploids on the mating plate. Following a 48 h growth at 30 ºC, colonies on each plate were counted and a linear regression was fit to this data. However, a single mating event may result in several observed diploids because some growth occurs on a mating plate, meaning that early mating events may be counted more than once. Thus, to generate a more conservative estimate of the mating efficiency, we divided the number of observed diploids by the fold increase in the number of cells on the mating plate (~ 1.7). This procedure is likely to be an underestimate of the true mating efficiency for two reasons: 1) it assumes that all diploids are generated before cell outgrowth, while it is likely that some are generated after one or more haploid cell divisions, and 2) it assumes that diploids undergo the same number of cell divisions as haploids, yet mating takes ~ 4 hours [18], meaning that haploids are likely to undergo more cell divisions during the outgrowth on the mating plate. Nevertheless, the lower bound of the mating efficiency reported here is the most useful measure for the ultimate scalability of the assay.

LoxP recombination efficiency estimation

A loxP recombination efficiency test was performed on four randomly picked clones from a pooled mating between iSeq-barcoded PCA strains (above). Each clone was grown in 5 ml YPD + Nat + Hyg liquid media for 24h at 30ºC, spun down, and resuspended into 3.2 ml of YPG liquid media at a cell concentration of ~2 x 10^8 cells/ml to induce Gal-Cre mediated loxP recombination. Cells were grown for 24 h at 30ºC, and a cell count was taken to calculate the fold increase in cells in the recombination media (~1.7-fold growth). Cells were plated at three densities (500, 1000, and 2000 cells/plate) on SC-Ura agar and incubated for 48 h at 30ºC. Each plate was counted and a linear regression was fit to this data to estimate the total number of recombinant cells. Similar to mating frequency estimations described above, a single recombination event may result in several observed recombinants because some growth occurs in the recombination media. Thus, to generate a lower bound of the recombination efficiency, we divided the number of observed diploids by the fold increase in the number of cells in the recombination media.

Supplementary Note 7: **Comparison between bulk and pairwise mating**

Pairwise mated libraries were sequenced at a higher depth than bulk mated libraries (~200 reads per barcode and ~67 reads per barcode, respectively). The compare barcode frequency distributions at similar read depths, we sampled pairwise mating reads (without replacement) to ~67 reads per barcode. Shown in Figure 5b is results from a typical sampling. Other sampling attempts did not significantly change the results or conclusions.

Supplementary References

1. Ghaemmaghami, S., Huh, W. K., Bower, K. & Howson, R. W. Global analysis of protein expression in yeast. *Nature* **425,** 737–741 (2003).
2. Levy, S. F. *et al.* Quantitative evolutionary dynamics using high-resolution lineage tracking. *Nature* **519,** 181–186 (2015).
3. Goldstein, A. L. & McCusker, J. H. Three new dominant drug resistance cassettes for gene disruption in Saccharomyces cerevisiae. *Yeast* **15,** 1541–1553 (1999).
4. Albert, H., Dale, E. C., Lee, E. & Ow, D. W. Site-specific integration of DNA into wild-type and mutant lox sites placed in the plant genome. *The Plant Journal* **7,** 649–659 (1995).
5. Zhang, Z. & Lutz, B. Cre recombinase-mediated inversion using lox66 and lox71: method to introduce conditional point mutations into the CREB-binding protein. **30,** e90 (2002).
6. Lee, K., Zhang, Y. & Lee, S. E. Saccharomyces cerevisiae ATM orthologue suppresses break-induced chromosome translocations. *Nature* **454,** 543–546 (2008).
7. Brachmann, C., Cost, G. & Boeke, J. Designer deletion strains derived from Saccharomyces cerevisiae S288C: a useful set of strains and plasmids for PCR-mediated gene disruption and other applications. *Yeast* **14,** 115—32 (1998).
8. Kao, K. C. & Sherlock, G. Molecular characterization of clonal interference during adaptive evolution in asexual populations of Saccharomyces cerevisiae. *Nature Genetics* **40,** 1499–1504 (2008).
9. Gietz, R. D. & Schiestl, R. H. High-efficiency yeast transformation using the LiAc/SS carrier DNA/PEG method. *Nat Protoc* **2,** 31–34 (2007).
10. Tong, A. H. *et al.* Systematic genetic analysis with ordered arrays of yeast deletion mutants. *Science* **294,** 2364–2368 (2001).
11. Pan, X. *et al.* A robust toolkit for functional profiling of the yeast genome. *Molecular Cell* **16,** 487–496 (2004).
12. Lindstrom, D. L. & Gottschling, D. E. The mother enrichment program: a genetic system for facile replicative life span analysis in Saccharomyces cerevisiae. *Genetics* **183,** 413–22– 1SI–13SI (2009).
13. Tarassov, K. *et al.* An in vivo map of the yeast protein interactome. *Science* **320,** 1465–1470 (2008).
14. Baryshnikova, A. *et al. Chapter 7 - Synthetic Genetic Array (SGA) Analysis in Saccharomyces cerevisiae and Schizosaccharomyces pombe. Guide to Yeast Genetics: Functional Genomics, Proteomics, and Other Systems Analysis* **470,** 145–179 (Elsevier Inc., 2010).
15. Blundell, J. R. & Levy, S. F. Beyond genome sequencing: Lineage tracking with barcodes to study the dynamics of evolution, infection, and cancer. *Genomics* **104**, 417-430 (2014).
16. Venkataram, S. *et al.* Development of a Comprehensive Genotype-to- Fitness Map of Adaptation-Driving Mutations in Yeast. Cell **166**, 1585–1596 (2016).
17. Huh *et al.* Global analysis of protein localization in budding yeast. Nature **425**, 686–691 (2003).
18. Bardwell, L. A walk-through of the yeast mating pheromone response pathway. *Peptides* **25,** 1465–1476 (2004).
